# Neutral Genomic Microevolution of a Recently Emerged Pathogen, *Salmonella enterica* Serovar Agona

**DOI:** 10.1371/journal.pgen.1003471

**Published:** 2013-04-18

**Authors:** Zhemin Zhou, Angela McCann, Eva Litrup, Ronan Murphy, Martin Cormican, Seamus Fanning, Derek Brown, David S. Guttman, Sylvain Brisse, Mark Achtman

**Affiliations:** 1 Environmental Research Institute, University College Cork, Cork, Ireland; 2 National Salmonella Reference Laboratory, Bacteriology Department, Galway University Hospital, Galway, Ireland; 3 University College Dublin Centre for Food Safety, School of Public Health, Physiotherapy and Population Science, Dublin, Ireland; 4 Scottish Salmonella, Shigella and Clostridium difficile Reference Laboratory, Microbiology Department, Stobhill Hospital, Glasgow, United Kingdom; 5 Centre for the Analysis of Genome Evolution and Function, University of Toronto, Toronto, Canada; 6 Institut Pasteur, Microbial Evolutionary Genomics Unit, Paris, France; Universidad de Sevilla, Spain

## Abstract

*Salmonella enterica* serovar Agona has caused multiple food-borne outbreaks of gastroenteritis since it was first isolated in 1952. We analyzed the genomes of 73 isolates from global sources, comparing five distinct outbreaks with sporadic infections as well as food contamination and the environment. Agona consists of three lineages with minimal mutational diversity: only 846 single nucleotide polymorphisms (SNPs) have accumulated in the non-repetitive, core genome since Agona evolved in 1932 and subsequently underwent a major population expansion in the 1960s. Homologous recombination with other serovars of *S. enterica* imported 42 recombinational tracts (360 kb) in 5/143 nodes within the genealogy, which resulted in 3,164 additional SNPs. In contrast to this paucity of genetic diversity, Agona is highly diverse according to pulsed-field gel electrophoresis (PFGE), which is used to assign isolates to outbreaks. PFGE diversity reflects a highly dynamic accessory genome associated with the gain or loss (indels) of 51 bacteriophages, 10 plasmids, and 6 integrative conjugational elements (ICE/IMEs), but did not correlate uniquely with outbreaks. Unlike the core genome, indels occurred repeatedly in independent nodes (homoplasies), resulting in inaccurate PFGE genealogies. The accessory genome contained only few cargo genes relevant to infection, other than antibiotic resistance. Thus, most of the genetic diversity within this recently emerged pathogen reflects changes in the accessory genome, or is due to recombination, but these changes seemed to reflect neutral processes rather than Darwinian selection. Each outbreak was caused by an independent clade, without universal, outbreak-associated genomic features, and none of the variable genes in the pan-genome seemed to be associated with an ability to cause outbreaks.

## Author Summary

Pulsed-field gel electrophoresis (PFGE) is the gold standard for recognizing outbreaks of food-borne disease. Comparative genomics has been used to elucidate transmission chains and evolutionary patterns within bacterial pathogens. However, their global genetic diversity has not yet been compared across multiple food-borne outbreaks. It was uncertain whether such outbreaks are caused by closely related organisms or are due to genetic properties that were recently acquired by horizontal genetic transfer. We investigated *Salmonella enterica* serovar Agona, a common cause of food-borne gastroenteritis, by sequencing genomes from bacterial strains from five outbreaks, as well as sporadic disease and the environment. Each outbreak was caused by a genetically distinct variant, but closely related bacteria were also found in the environment or in animal sources. The erroneous identity of bacteria according to PFGE reflected repeated, neutral acquisitions of bacteriophages, and genetic acquisitions or losses were not uniform across outbreak strains. Our results indicate that genomic differences used by epidemiologists to reach important decisions for public health can be stochastic and unrelated to disease or fitness. Our results also define the population structure and minimal age of Agona, and suggest that many serovars of *Salmonella* may be of recent origin.

## Introduction

**Figure 1:**
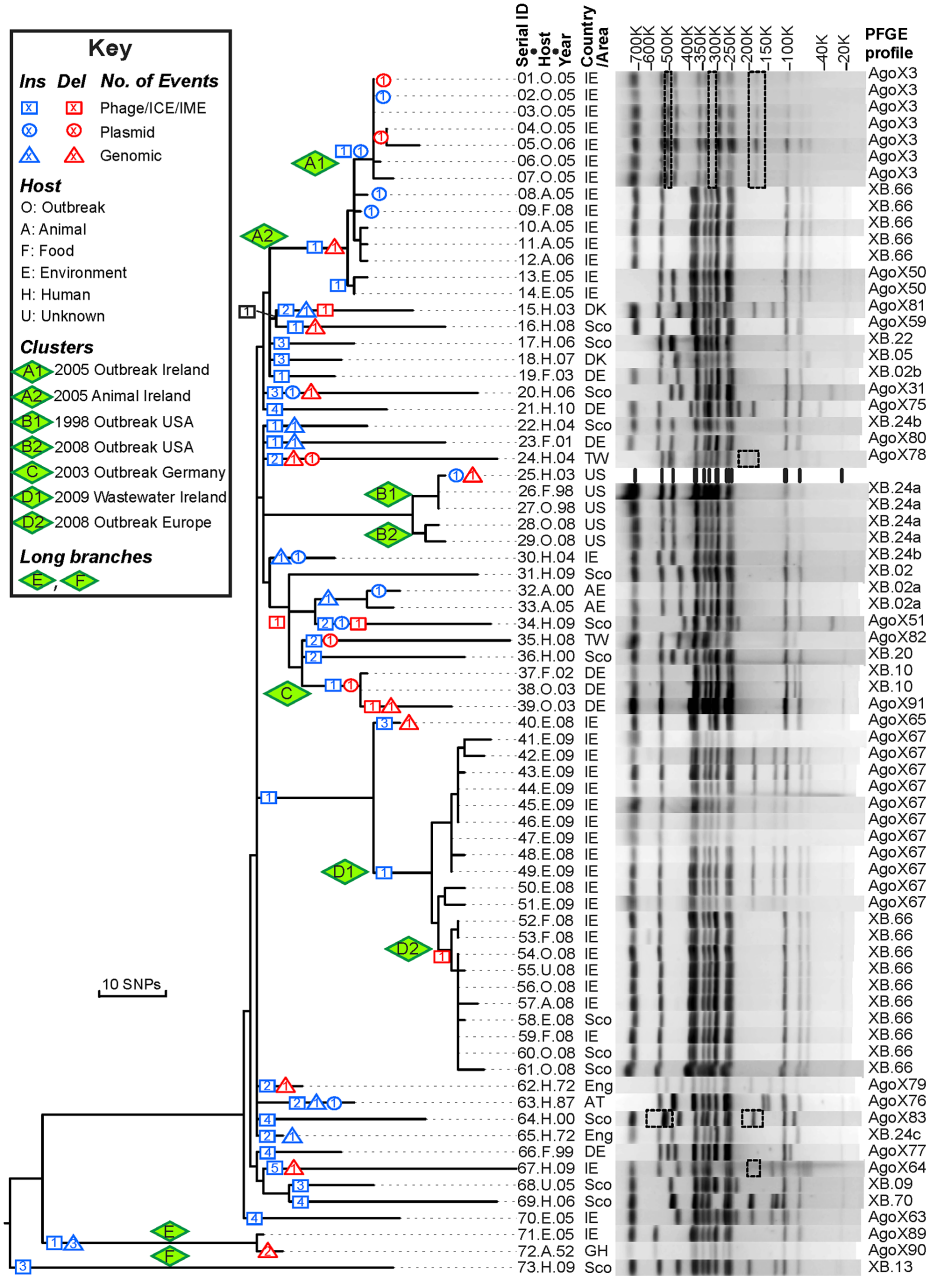
Genealogy of 73 Agona genomes based on SNPs and indels *versus* PFGE patterns. Left: Maximum parsimony genealogy inferred from 846 non-homoplastic, non-recombinant, non-mobile, non-repetitive core SNPs. The numbers of independent insertions or deletions of mobile elements (Figure S1, Dataset S1) are indicated by symbols at the nodes within the genealogy (Key). Isolates that are related to outbreaks were clustered in clades A–D and their sub-clades (A1, A2, *etc.*). Centre: Strain designations include a serial number, an abbreviation for Host and the year of isolation (Dataset S2). Right: PFGE patterns and profiles of *XbaI*-digested DNA for each strain except for the reference genome (25.H.03 [SL483]), which was predicted *in silico*. All observed PFGE bands were also predicted from genomic analyses except for bands in dashed boxes. The detailed sizes and existence of each band are listed in Dataset S3.

In 2008, *Salmonella enterica* serovar Agona (henceforth Agona) caused an outbreak associated with ready to eat meat that was distributed from Ireland to seven European countries, infecting at least 163 individuals with two deaths [1]. We were intrigued by the 2008 outbreak because all Agona isolated from either contaminated food or infected individuals shared an indistinguishable *XbaI* PFGE pattern (SAGOXB.0066; henceforth XB.66) whereas isolates from a waste water source in the food plant exhibited a related PFGE pattern, AgoX67, that differed by two bands (Figure 1). XB.66 Agona were also isolated three years earlier (2005) from infected pigs and poultry in Ireland, but contemporary human isolates exhibited a very distinct PFGE pattern (AgoX3). Similar observations have been reported from other outbreaks of Agona [1]–[3] and other *S. enterica* serovars. From a public health perspective, identical PFGE patterns have been considered to mark clonal expansions of a single, source-specific strain [4], especially when epidemiological investigations provide supportive evidence [5]. However, a single PFGE pattern can represent multiple bacterial strains [5], [6]. Different PFGE patterns are usually thought to represent unrelated bacteria, except that variation in a few bands seems to occur with time during prolonged outbreaks [5]. In some organisms, continued or repeated outbreaks have been attributed to the existence of epidemic clones with broad distributions [7]–[9], which may have arisen due to novel genomic islands that were imported by horizontal gene transfer (HGT) [8] or due to SNPs that cause antibiotic resistance [10]. It thus seemed possible that the Agona outbreak might have represented the evolution of such an epidemic clone. However, the genomic basis for differences in PFGE patterns within serovars of *S. enterica* has not been determined. More specifically, what are the genomic characteristics that result in diverse PFGE patterns, and how closely do PFGE patterns reflect genetic relationships? Secondly, it is not clear whether epidemic clones generally arise through HGT or selective pressures, or simply reflect the random expansion of particular clades through neutral genetic drift.

Agona is particularly suitable for addressing these questions because multilocus sequence typing indicates that it is genetically monomorphic [11]. All Agona isolates belong to only four closely related sequence types, which form a single eBurst Group, eBG54. This is comparable to the genetic diversity found with serovar Typhi [12], [13], and much more uniform than many other serovars of *S. enterica*, such as Montevideo, Typhimurium or Newport, which each encompass multiple eBGs [11], [14]. The limited diversity of Agona might even reflect a recent origin, especially because Agona was first isolated from cattle in Ghana in 1952 [15]. Agona became one of the common global causes of salmonellosis of animals and humans [11], [16], [17] after the late 1960's, when it was imported to the USA and Europe in contaminated fishmeal from Peru [18], [19]. Today, Agona is a common contaminant of livestock and vegetables in farms [20]–[22], as well as prepared food from food factories [1], [23]–[25]. In addition to the Irish outbreak, it has caused multiple outbreaks of food-borne gastroenteritis in the UK [3], USA [24], [26], Finland [27], France [25] and Germany [28]. Thus, Agona provides the opportunity for investigating whether isolates from outbreaks differ from sporadic isolates from a variety of hosts and geographical sources, and whether measureable evolution has occurred over a period of several decades.

Here we present a genomic analysis of a global collection of Agona, including isolates from five outbreaks, and compare the diversity introduced *via* mutation, homologous recombination and mobile elements with the phylogeographical and PFGE patterns.

## Results/Discussion

In order to explore its genetic diversity, we performed short-read genomic sequencing of 72 isolates of serovar Agona (Dataset S2, Table S1). These included 38 isolates from outbreaks in the USA in 1998 and 2008 [24], [26], Germany in 2002–2003 [28] and the multi-country outbreak originating from Ireland in 2008 (XB.66 PFGE pattern) [1]. We also included Irish isolates from 2005 (XB.66: cattle and poultry; AgoX3: humans) and 2008/2009 (AgoX67; food plant waste water). As controls, we sequenced the genomes of 18 isolates with diverse PFGE patterns from Ireland and Scotland, and 16 other isolates from diverse geographic historical sources and dates of isolation, including the first Agona strain from 1952. We also included the reference genome of strain SL483 (here 25.H.03) for a total of 73 genomes.

**Figure 2:**
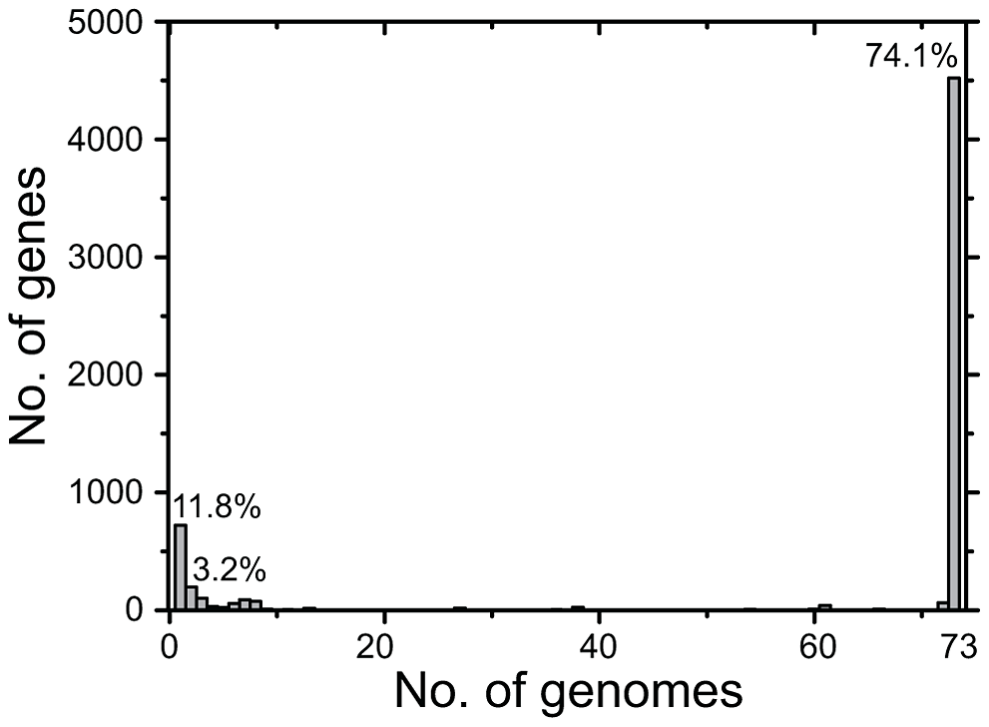
Number of CDSs *versus* number of genomes in which they were present. All CDSs in the pan-genome were used for these calculations, except that repetitive CDSs such as IS elements were each counted as one CDS.

Many prior genomic analyses of genetically monomorphic genomes have concentrated on SNPs in the core genome [13], [29], [30], which are easy to call and simple to interpret. Often this is performed by mapping reads against a reference genome [30]–[32], which excludes regions of greater diversity and also excludes the accessory genome. Alternatively, SNP calling can be performed by mapping assemblies against a reference genome [33], which faces the problem that *de novo* assemblers can miscall the consensus nucleotide when they do not use the consensus number of reads showing the same nucleotide. We followed a different strategy to ensure that we obtained accurate SNP calls as well as capturing recombinant regions of high SNP density and variation in the accessory genome. We *de novo* assembled and annotated genomes from our data, and remapped all reads against their own assembly to confirm the consensus nucleotides in each assembly. In multiple genomic projects that we are currently investigating, this strategy has eliminated numerous false nucleotide calls due to a mis-called consensus and elucidated regions of high SNP density which were previously unmapped. We then identified a 4.27 MB core genome by comparing all 73 genomes, which was reduced to 4.22 MB after filtering against repetitive or mobile DNA (Table 1). The accessory genome comprised approximately 510 Kb for each isolate (Figure S2), and included 1,582 CDSs spanning a total of almost 1.3 MB over all genomes (Figure 2, Table S2). We re-sequenced genomes from 10 strains a second time, and confirmed that they were indistinguishable from the first set (Dataset S2).

**Table 1:** Features of the *S. enterica* Agona non-repetitive non-mobile core genome.

	Number
Genomes	73
MLST STs	13, 1215, 1328
Mean read coverage (range)	170 (36–1464)
Total genome length	4,782,841±58,946 bp
Core genome	4,271,427 bp
Core genome minus repetitive/mobile elements	4,224,687 bp
CDSs	4,520
SNPs/indels	4,013/118
Mutational SNPs/indels	846/99
Homoplastic SNPs/indels	3/1
Recombination events/length	42/360 Kb
Recombinational SNPs/indels	3,164/18

**Figure 3:**
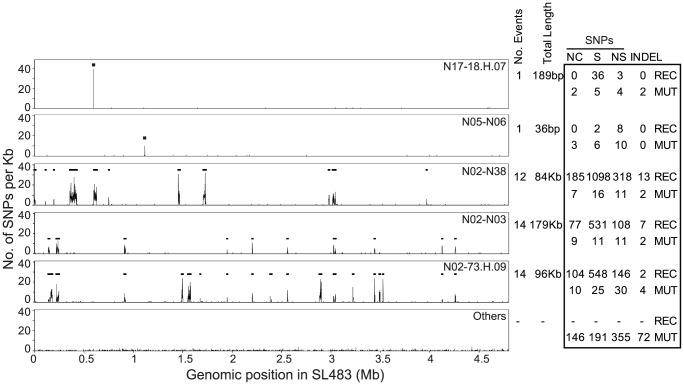
Recombinant regions according to ClonalFrame. Left: Recombinant segments in five genealogical nodes are indicated as horizontal black lines spanning regions with high SNP density. The lowest plot (Others) indicates the average SNP density in all other nodes. Right: Numbers of non-coding (NC), synonymous (S), non-synonymous (NS) SNPs, and indels, subdivided into recombinational (REC) and mutational (MUT) events.

Nucleotides in each core genome were used to identify SNPs against the reference genome with the help of nucmer (MUMmer package). Most SNPs were assigned by ClonalFrame [34] to 42 regions of higher SNP density that had been introduced by homologous recombination (Figure 3; see below). 849 other SNPs (Dataset S4) were used to derive a preliminary genealogy of the 73 genomes for filtering homoplasies. All but three of these 849 SNPs arose only once within that genealogy, indicating that they reflected individual mutational events. Two of the three other, homoplastic SNPs were located next to each other in a gene of unknown function, and the third represented two independent mutations in a fucose isomerase gene (Table S3). All three homoplastic SNPs were excluded from further analyses because homoplasies arise from Darwinian selection or mutational hot-spots, which do not follow the same molecular clock rate as other mutations, and are inconsistent with neutral genealogies. The remaining 846 SNPs were used to generate a final, completely parsimonious mutational genealogy, with 143 nodes (Figure 1, Figure S1), that was used for historical reconstructions and dating analyses. The same genealogy was obtained by maximal parsimony and maximum likelihood. The root of the genealogy, corresponding to the most recent common ancestor (MRCA) of all Agona, was inferred from comparisons of all 846 SNPs against four genomes from other serovars of *S. enterica*. 805 nucleotide variants at the inferred root were found in one or more of these other serovars, consistent with those variants being ancestral. The ancestral nucleotide for the 41 other positions was assigned to the variant predicted by the rooting position in the genealogy, ignoring contradictory data for four nucleotide variants.

The 4.2 Mb, non-recombinant core genome also contained at least 100 indels of up to 15 bp in size (Dataset S4). (We provide only a minimal number of indels because none of the available algorithms for detecting indels in short read genomic sequences is fully reliable [35]). Of these 100 indels, only one was homoplastic, and the others were scattered throughout the SNP genealogy, or in terminal nodes (Figure S3). Thus, the genealogy of indels supports the SNP genealogy, or is at least consistent with it. The indels were relatively abundant in intergenic regions (37 indels) or did not change the reading frame of coding regions (14) because they involved multiples of three nucleotides. However, 49 indels result in frameshifts.

### Agona Is a Recently Emerged Pathogen

The mutational genealogy consists of three distinct branches (Figure 1, Figure S1). 70 of the genomes fell into one main branch, including both isolates from 1972 as well as all outbreak isolates. The isolates from outbreaks clustered by date and geography into clades A (Ireland 2005), B (USA 1998, 2008), C (Germany, 2002/2003) and D (Ireland and other countries, 2008/2009). Further clade subdivisions are described in greater detail below. A second, long branch (E) contained the 1952 isolate from Ghana plus an Irish isolate from 2005, and a third, long branch (F) contained one isolate from Scotland in 2009.

**Figure 4:**
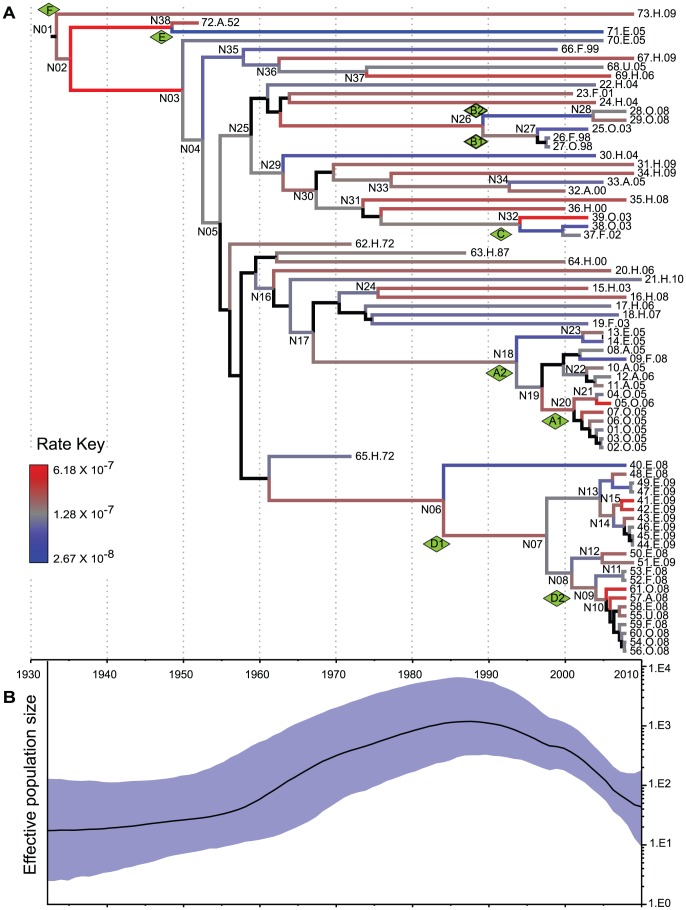
Reconstruction of MRCA dates and changes in population size. Analyses were based on analyses of 846 non-recombinant, non-repetitive, non-mobile concatenated core SNPs by the relaxed GMRF model in Beast v1.7.1 [36]. (A) A maximum clade credibility tree. Branch colors indicate substitution rates transformed to a logarithmic scale (Rate Key), except that posterior probabilities of <0.5 for nodes and branches are indicated in black. (B) Bayesian skyride plot showing changes in effective population size of serovar Agona over time (black line) with the extent of the 95% confidence intervals shaded in blue.

The root of the genealogy corresponding to the MRCA of all Agona is located between branch F and the other two branches. The numbers of SNPs from the MRCA within individual genomes increased linearly with their date of sampling (Figure S4, *R* ^2^ = 0.3), indicating that Agona has evolved over the time of sampling. This linear regression indicates that modern Agona originated about 1900, or earlier. A more sophisticated Bayesian analysis (Beast) indicated that the MRCA evolved in or before 1932 (CI95%: 1918–1945) (Table S4, Figure 4A). This analysis used a GMRF model, which allows fluctuations in population size, with a uncorrelated log-normal relaxed clock [36]. According to a skyride plot (Figure 4B), Agona underwent a major population expansion from the 1950's to the 1980's, in agreement with the epidemiological data, according to which Agona first became common in the USA and Europe in the late 1960's [18], [19]. An alternative relaxed clock model with constant population size yielded a slightly better fit (higher Bayes factor) than the GMRF model, and a date for the MRCA of 1799 (CI95%: 1618–1928) (Table S4). However, we place greater trust in the calculations from the GMRF model because the rapid, global expansion in the 1960's should be coupled with an increment in effective population size [37], [38]. A third model based on a strict molecular clock with constant population size resulted in markedly poorer Bayes factor scores (Table S4), and can be discounted. Visual examination of the data also argues against a strict clock rate because genomes within clade B and branch E had hardly diverged during 10 and 53 years, respectively.

These calculations indicate that Agona is a recently evolved pathogen, which likely arose about 80 years ago. Consistent with this interpretation, Agona was first identified in 1952. Currently, more than 1,500 serovars have been defined within *S. enterica* subsp. *enterica* [39]. Some of these serovars, such as Typhi, Enteritidis and Typhimurium, had already been isolated by the late 19^th^ century, and are almost certainly older than Agona. Indeed, Typhi was previously estimated to have evolved 10,000–50,000 years ago [12]. However, that calculation was based on a very slow clock rate that has since been discredited [40], and the age of Typhi and most serovars is currently unknown. The number of known serovars has increased dramatically since World War II, which has been attributed to the increased importation of food products from global sources to Europe and the USA [41]. Alternatively, many serovars may have evolved in the 20^th^ century, especially because multiple other eBGs are also genetically monomorphic [11].

Not all genetically monomorphic serovars are necessarily recently evolved because molecular clock rates can differ by several orders of magnitude between different bacterial species [40], and even vary within a single species over differing time periods (Table S5). According to our calculations, the mean clock rate for the accumulation of core SNPs in Agona was 9.3×10^−8^ per nucleotide per year (CI95%: 5.7×10 ^−8^–1.3×10^−7^) (Table S5), and the extremes of the clock rate for different branches within the genealogy spanned a 23-fold range (Figure 4A). The Agona clock rate is among the slowest bacterial intra-species clock rates that have been estimated (Table S5). We note that some differences in mutation rates can also reflect the period of sampling because clock rates slow down over time due to purifying selection of slightly deleterious mutations [40], [42], [43]. A mutation rate (7×10^−5^) that is 700-fold faster than that of Agona has been reported for a clade of serovar Montevideo with a single PFGE pattern [44]. That mutation rate cannot be directly compared with the rates summarized in Table S5 because they reflect mutations per nucleotide per year whereas the Montevideo calculation was based on mutations per variable site per year.

### Homologous Recombination within the Core Genome

**Figure 5:**
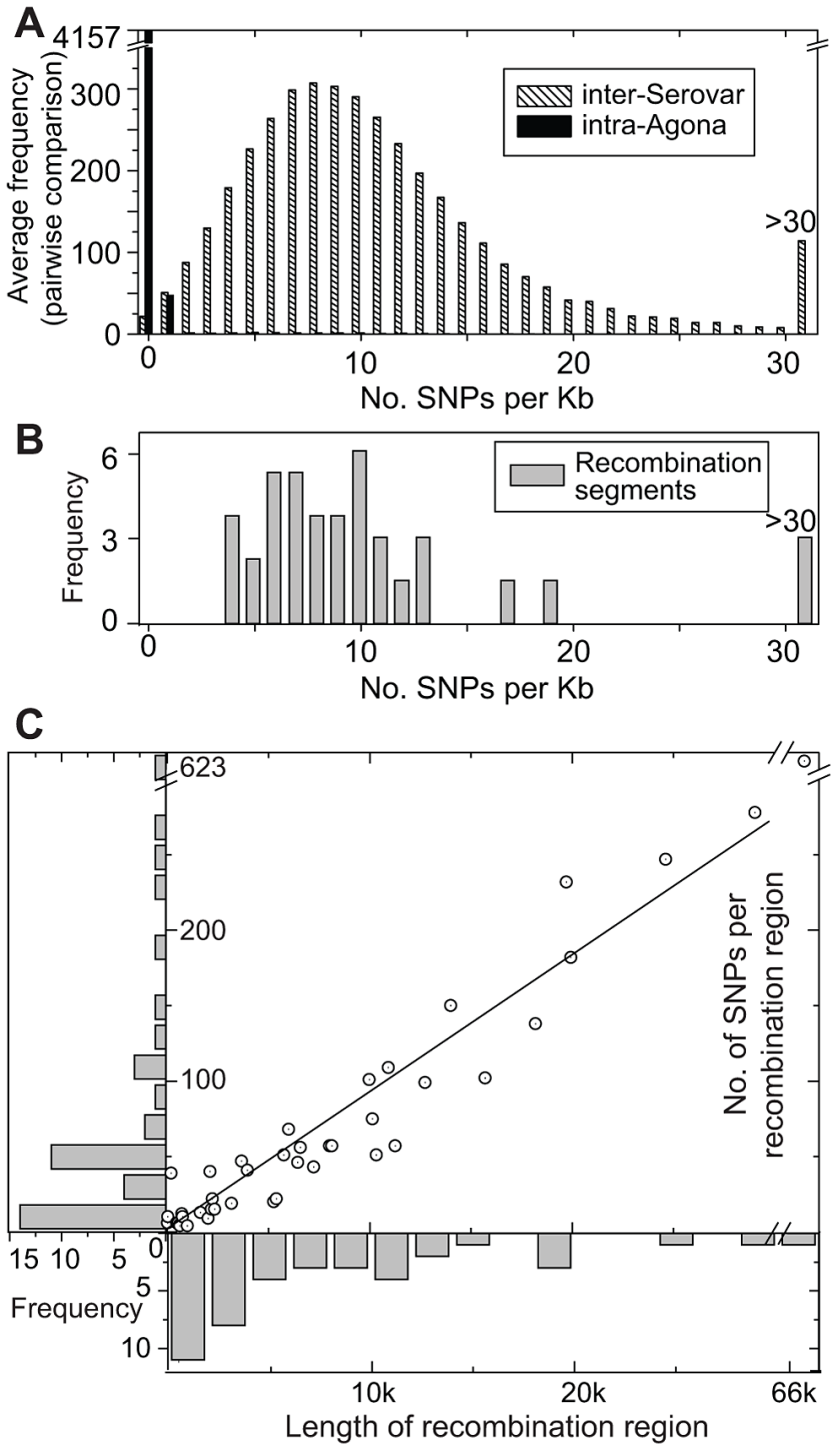
SNP densities in recombinant and non-recombinant regions of the Agona core genome. (A) Average frequency of SNPs per Kb from pair-wise, 1 Kb sliding window comparisons between the genome of Agona SL483 *versus* 12 genomes from serovars Choleraesuis, Dublin, Enteritidis, Gallinarium, Heidelberg, Newport, Paratyphi A, Paratyphi B, Paratyphi C, Schwarzengrund, Typhi, and Typhimurium (hatched boxes). A comparable distribution of mean pair-wise diversity between all 73 Agona genomes is indicated by black boxes. (B). SNP density in recombination segments. (C) Frequency distributions of the length of recombinant segments (x-axis) and number of SNPs per recombinant region (y-axis). Each recombination region is indicated by an open circle with an internal dot. The linear regression of these data indicates that 9.2 SNPs are found per recombinant Kb (*R* ^2^ = 0.97).

Our analyses identified 42 regions in the non-mobile core genome that we attributed to homologous recombination because of high SNP density (Figure 3, Table S6). These regions were probably imported from other serovars of *S. enterica* because their SNP densities overlapped with the SNP densities in comparisons of Agona versus 12 genomes from other serovars (Figure 5). In contrast, SNP densities in pair-wise comparisons of the non-mobile core genome of Agona were much lower. The 42 recombination events (360 kb) introduced 3,164 SNPs and 18 indels (Table 1, Dataset S5), *versus* only 849 SNPs and 100 indels in the 4.2 Mb core genome that we attribute to mutations. However, the recombination events were only found in five of the 143 nodes within the mutational SNP genealogy (Figure 3), indicating that homologous recombination was a rare event. Three of the five nodes are the basal nodes of the three primary branches in the genealogy, each of which differs by multiple protein sequence variants due to recombination (Dataset S5). These proteins include enzymes, transcriptional regulators and potential virulence factors (Dataset S6). The acquisition of these recombinant segments may have been important for the fitness of these branches and their ability to exist over decades, or at least to out-compete other putative competitors which are not included in our sample. The two other nodes were at the base of cluster D and within a singleton (sporadic) genome. Each had only one recombinant region, and additional recombinant regions were not found within the three other outbreak clusters, nor among the other sporadic isolates. Thus, the ability to cause an outbreak was not generally associated with the acquisition of genetic variants by homologous recombination.

### Non-Mutational Genetic Diversity in the Accessory Genome

CDSs in the accessory genome were assigned to IS elements, bacteriophages, plasmids, integrative conjugative elements (ICE) and integrative mobile elements (IME), and other genomic islands (Table 2). These variably present CDSs were scored as representing introductions or deletions (indels) relative to the MRCA. All accessory CDSs were clustered in regions of two or more CDSs except that the majority of the IS elements contained only one CDS. Exceptionally, two other singleton CDSs were identified (SAPA_G0518, a LysR substrate binding domain protein, and SAPA_G0527, an O-antigen polymerase) (Dataset S1, S7), which had been deleted from one or two genomes, respectively. Most of the accessory CDSs corresponded to introductions into only one or two genomes and almost all others were introductions that were only found in three to eight genomes (Figure 2).

**Table 2:** Multiple introductions and deletions of mobile elements.

		Number		
Closest relative	Type (Number)	Introductions	Deletions	Sites (tRNA/ncRNA)
T4SS effector	Genomic island (1)	0	2	1
O-antigen polymerase	Genomic island (1)	0	2	1
p58	Plasmid (1)	1	3	NA
pO157_Sal (IncI)	Plasmid (2)	2	0	NA
SGI-1A/SGI-1C	IME (2)	2	0	1
ICESe4	ICE (2)	2	0	2 (2)
IS21 family	IS element (2)	2	0	2
Gifsy-1	Bacteriophage (3)	3	0	2
P4	Bacteriophage (4)	4	0	3 (3)
IS1 family	IS element (2)	4	0	2
IS66 family	IS element (3)	4	0	3
IS5 family	IS element (3)	4	1	4
IS6 family	IS element (1)	4	2	1
Antibiotic resistance-plasmid (IncI1)	Plasmid (4)	5	2	NA
IS3 family	IS element (4)	5	2	4
Rhs protein	Genomic island (1)	5	3	1
IS4 family	IS element (1)	7	0	7
Lambda	Bacteriophage (5)	8	1	3 (2)
Seroconverting phage	Bacteriophage (9)	9	0	2 (1)
Fels-2	Bacteriophage (11)	17	2	2 (1)
Tn3 family	IS element (8)	19	3	8
P2	Bacteriophage (16)	25	2	5 (2)

Each introduction or deletion in a node of the genealogy is counted only once, even if that introduced mobile element is present in multiple descendent genomes. The number of distinct sequence variants within each category is indicated by (Number) after Type. Where mobile elements integrated into multiple locations within the genome, each such integration was associated with a distinct sequence variant (Dataset S1), and they represent independent insertions. Additional, unrelated non-IS mobile elements were each introduced on a single occasion (Dataset S1), consisting of 2 ICE/IMEs, 3 bacteriophages, 3 plasmids and 4 other genomic islands, for a total of 95 introductions of non-IS mobile elements. Five other genomic islands were each deleted once, for a total of 22 deletions of non-IS mobile elements. Similarly, five IS elements were introduced on one occasion each (Table S8, S9), for a total of 54 introductions.

In contrast to the rare homologous recombination in the core genome, the 1.3 Mb accessory genome contains an extraordinarily high level of genetic diversity (Table S2; Dataset S1). These indels included 54 introductions and 8 deletions of IS elements, predominantly of the IS3 and Tn3 families, as well as 95 introductions and 22 deletions of other mobile elements (Figure 1, Figure S1, Table 2, Table S2; Dataset S1). Multiple P2-like, Fels-2-like bacteriophages and lambda-like bacteriophages were each introduced on multiple occasions to one or more genomic locations, and occasionally subsequently lost. This observation can potentially explain the moderate diversity in lysogenic bacteriophages that was found by phage typing in the 1980's [16]. We also found frequent independent introductions of a genomic island encoding an Rhs protein and a type VI secretion system, a sero-converting bacteriophage, IncI1 antibiotic resistance plasmids and P4-like and Gifsy-1-like bacteriophages. Similarly, we observed multiple, independent loss events of a p58-like plasmid, the Rhs genomic island as well as two other genomic islands, and IncI1 antibiotic-resistance plasmids.

Former publications have stressed the potential of such genomic flux for introducing virulence factors, and the importance of genomic flux for adaptation to novel environments or hosts [45]–[48]. For example, multiple HGT events are associated with the USA300 MRSA lineage [7], a conjugative transposon has been independently acquired by two epidemic lineages of *Clostridum difficile* [49], and cargo genes on bacteriophages, insertion elements and virulence plasmids are thought to be responsible for the virulence properties of EHEC *Escherichia coli* [50]. Similarly, the CTX toxin was introduced to epidemic *Vibrio cholerae* as a cargo gene within a bacteriophage [51] and the hyper-virulence of a clade of M3 *Streptococcus pyogenes* was attributed to the acquisition of a bacteriophage [52]. However, those studies did not compare patterns of acquisition and loss of mobile elements with a detailed genealogy of strains of multiple related clades isolated globally from sporadic infections and outbreaks over decades.

We searched for virulence factors that might be associated with the acquisition of mobile elements in Agona, but only identified the *gogB* gene within Gifsy-1 bacteriophage, which was present in one genome from sporadic disease. We also searched for the gain of antibiotic resistance, and identified diverse genes encoding antibiotic resistance within two chromosomal integrons (SGI-1A, SGI-1C element) as well as within the IncI1 antibiotic resistance plasmids (Dataset S7). Those mobile elements were also only found within sporadic isolates, except that one of the four IncI1 plasmids was present in a few antibiotic-resistant isolates from cluster A [1]. A copper resistance gene was present in a chromosomal CTnscr94 ICE element, again in a sporadic strain. Thus, the acquisition of virulence factors and antibiotic resistance genes was not routinely associated with outbreaks. Similar conclusions have been reached for *Streptococcus pneumoniae* from multiple global outbreaks [53].

**Figure 6:**
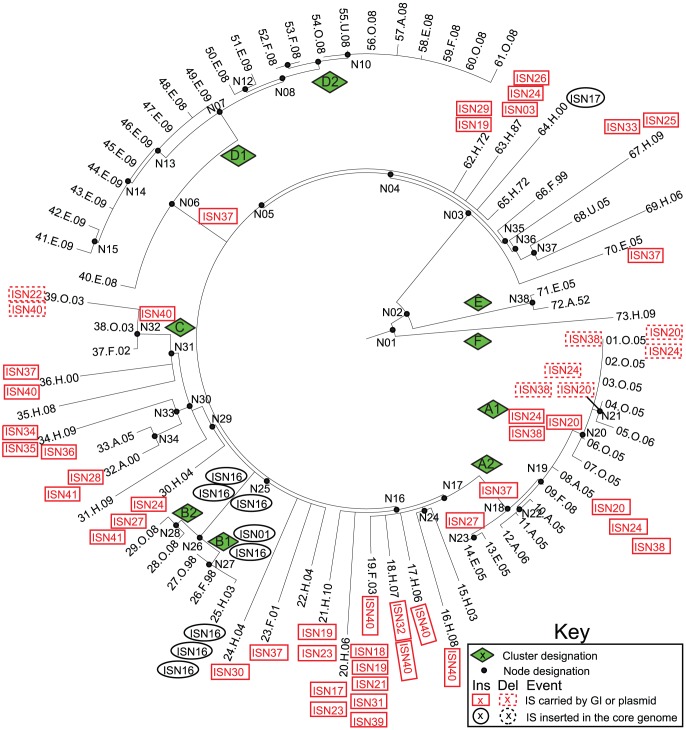
Insertions (solid lines) and deletions (dashed lines) of IS elements (Tables S7, S8, S9) in mobile elements (red boxes) or the core genome (black ellipses) mapped on a SNP genealogy of 73 Agona genomes. The SNP genealogy is based on a maximum parsimony tree based on 846 non-recombinant, non-mobile core SNPs (as in Figure 1), but drawn in radial fashion (Mega) for convenience. The tips of the branches include strain ID numbers, as in Figure 1 and Dataset S2. Node, clade and branch designations are also according to Figure 1 and Dataset S2.

We identified 41 distinct IS elements in the pan-genome (Tables S7, S8, S9). Of these, 27 IS elements varied in presence and/or copy number between genomes, and 25 of those were associated with mobile genetic elements. However, these too did not seem to be causally associated with a general ability to cause outbreaks, but rather seemed to represent genetic noise. The MRCAs of clades A and D had acquired ISN37 together with a Fels-2-like bacteriophage but clades B and C had acquired other clade-specific IS elements (Figure 6). Within the two sub-clades where multiple isolates were tested, sub-clades A2 and D1, the same IS elements were present in isolates from outbreaks and in closely related non-outbreak isolates. The MRCA of sub-clade A1 had acquired ISN20, ISN24, and ISN38 together with a plasmid, which was lost from three isolates within that subclade. The MRCA of clade B had acquired three copies of IsN16 (ISVsa5), which expanded to four copies in sub-clade B1 and seven in one isolate of that sub-clade (Figure 6). The acquisition and loss of IS elements was also common among isolates from non-outbreak clades.

The sequences and order of CRISPR spacers in CRISPR1 and CRISPR2 were previously shown to be identical in 11/12 Agona isolates [54] and the 12^th^ isolate had deleted five spacers. Our results were consistent with those observations, with the following rare exceptions (Dataset S8): the Agona MRCA contained a novel CRISPR2 spacer (AgoB15), which was deleted at the base of the main branch, and sporadic isolates had individually lost one of four spacers within CRISPR1 and one spacer within CRISPR2 (Dataset S8, Figure S5). Firstly, these results show that changes in CRISPR pattern are not specifically associated with outbreaks. Secondly, given the enormous diversity in content of bacterial prophages within the 72 genomes, it might have been expected from observations with environmental Archaea [55] that the CRISPR contents would vary dramatically after each acquisition of a novel lysogenic prophage. However, the CRISPR contents were highly uniform over the 60 years spanned by our strain collection.

Finally, we searched for cargo genes in the accessory genome that might be relevant to adaption or be under selection. Our annotated accessory genome contained 67 CDSs or clusters of CDSs that might fulfill these criteria, including CDSs that encode antibiotic resistance (Dataset S9). However, once again there was no correlation between the acquisition of cargo genes and outbreak clades (Figure S6). The MRCA of clade A lost the Rhs genomic island (GI9) and multiple clade A isolates acquired an IncI antibiotic resistance plasmid (P3A). None of the indels of putative cargo genes were specific for clades B, C, or D.

### Darwinian Selection *Versus* Neutrality

**Figure 7:**
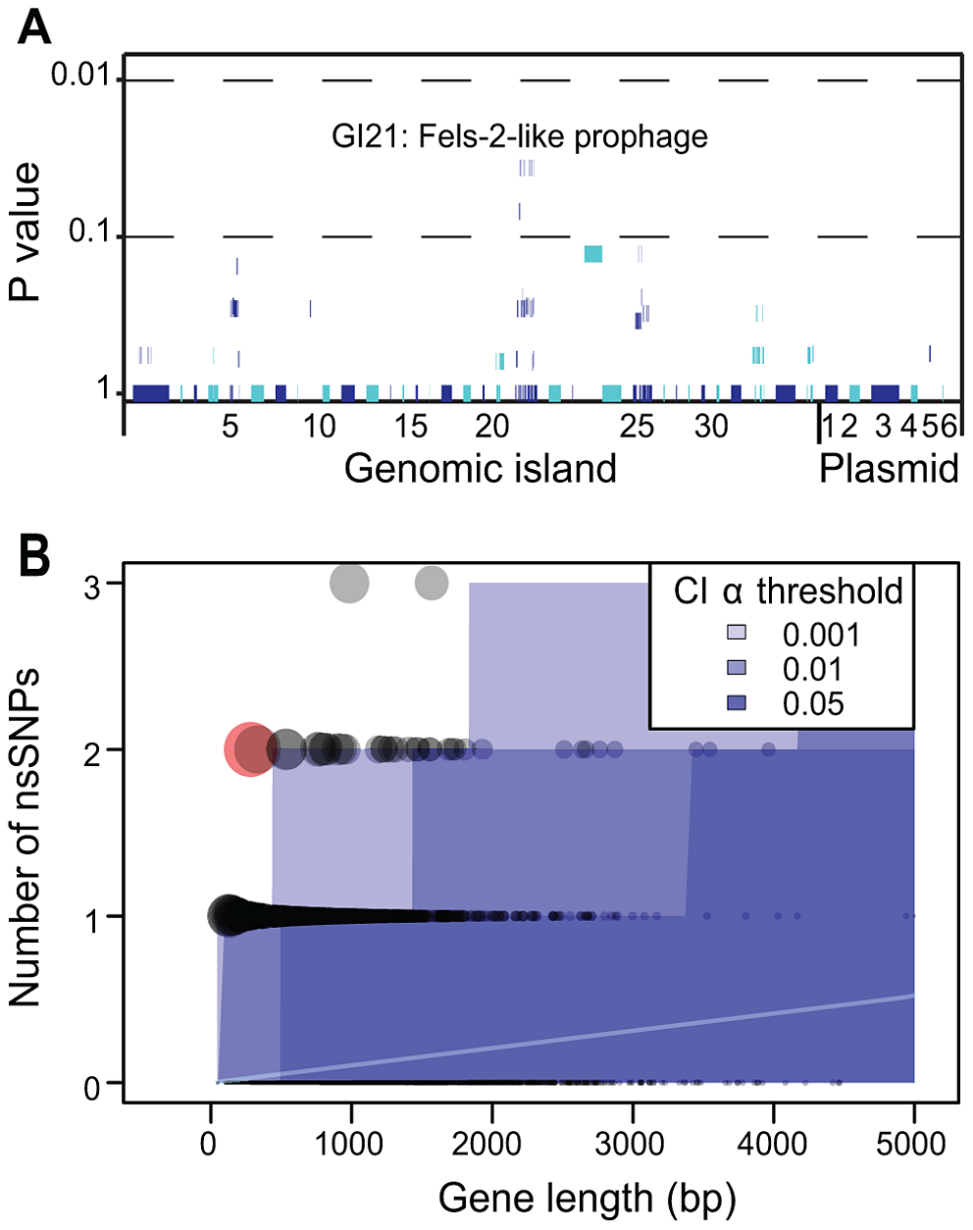
Lack of association of CDSs in the accessory genome with outbreak clades and statistical test of neutrality of non-synonymous mutations in the core genome. A) Likelihood of association of individual CDSs within the accessory genome with outbreak clades. Each of the 79 genomic islands and plasmids is indicated by alternating light and dark blue segments consisting of one vertical line per CDS. Individual genomes were assigned to one of four outbreak clades or one of 28 non-outbreak clades. Only eight CDSs within GI21 were significantly associated with all outbreak clades (Fisher exact test, p = 0.032). After a Bonferroni correction for multiple tests, no CDS was significantly associated with outbreak clades. B) Numbers of non-synonymous mutations per CDS in the non-recombinant, non-repetitive core genome as a function of gene length whose mean expectations are indicated by an internal white line. Each gene is represented by a circle, whose size is proportional to the deviation according to a χ^2^ statistic from theoretical expectations of a non-parametric test (Figure S5). Shades of blue indicate different α-thresholds (0.05, 0.01, 0.001) of the confidence intervals of the theoretical expectations, where 0.05 indicate CI 95%. The sole outlier identified in Figure S7B is indicated in red.

As just described, we did not detect any uniform importation or loss across outbreak clades of virulence factors, antibiotic resistance genes, IS elements, CRISPR spacers or other cargo genes. We then examined whether any CDS in the accessory genome might show an association with outbreak clades. These analyses identified eight CDSs with a significant correlation within GI21, a Fels-2-like bacteriophage (Figure 7A), but even these were insignificant after a Bonferroni correction for multiple tests. All but one isolate from outbreak clades A, B, C and D contained a clade-specific Fels-2-like bacteriophage, but with one exception (described below), these phages differed in sequence between the clades. This association between outbreak clades and a Fels-2-like bacteriophage is also not statistically different from neutral expectations because 18/28 non-outbreak clades also contained a Fels-2-like bacteriophage (Fisher exact test, *p* = 0.28). Similarly, possession of a Fels-2-like bacteriophage was not associated with disease because all isolates from environmental sources contained such a bacteriophage *versus* 42/54 isolates from infected humans, animals or food (*p* = 0.06).

We also searched for signs of Darwinian selection according to the frequency of non-synonymous mutations in individual CDSs within the non-recombinant, non-mobile, non-repetitive core genome. The *d*N/*d*S ratio, ω, was 0.67, indicating a general lack of positive (Darwinian) selection and only limited purifying selection. Similar results have been reported with multiple genetically monomorphic clades, including *S. enterica* serovar Typhi [13], ST239 *S. aureus* [56] and *Yersinia pestis* [57], [58]. However, ω is a crude estimator of Darwinian selection, especially when applied at the genomic level. We therefore examined the frequency of non-synonymous mutations per CDS in parametric and non-parametric tests that account for the length of each CDS and the expectations of a neutral frequency distribution (Figure S7). Only one CDS had a significant excess of non-synonymous mutations over neutral expectations (Figure 7B) due to two non-synonymous mutations within a length of 282 bps. This CDS (SeAg_B2298) encodes a hypothetical protein of unknown function. We conclude that, similarly to the accessory genome, there are no significant traces of Darwinian selection for genetic diversity within core CDSs. And because outbreaks were associated with multiple genetically distinct clades within the main branch, there was no association of the ability to cause outbreaks with any specific clade.

**Figure 8:**
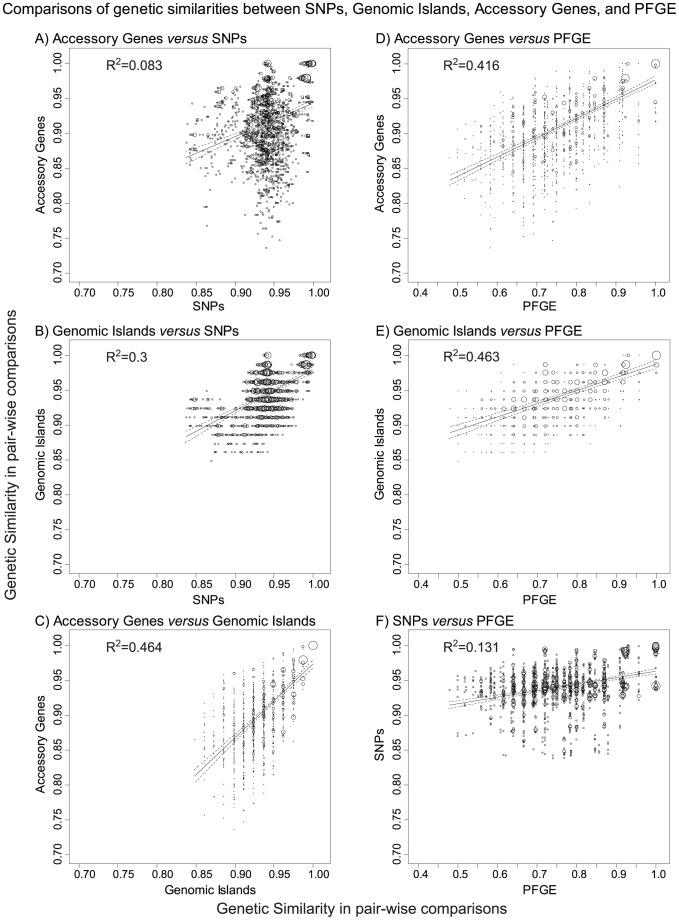
Comparisons of genetic similarities between core, non-repetitive, non-mobile SNPs, genomic islands, accessory genes, and PFGE bands. A–F) Plots of the frequencies of pairs of similarity scores for two matrices as indicated above each subplot. Matrices of the pair-wise genetic similarities were generated for UPGMA trees of the 72 new genomes in Bionumerics 6.5, and correspond to the interpretations provided in Dataset S1 (genomic islands), Dataset S3 (PFGE bands), Dataset S4 (SNPs), and Dataset S7 (accessory genes). The frequency of each pair of similarity values within a pair of matrices is indicated by circle size. A linear regression (solid line) with 95% confidence intervals (dotted lines) is shown for each pair of matrices as well as the coefficient of determination (*R* ^2^). All comparisons were statistically significant in Mantel tests of the paired matrices, with *p*≤10^−4^ except for part A, where *p* = 5×10^−4^.

One explanation of our observations might be that the main branch possesses specific mobile elements that support an ability to cause outbreaks by any of its sub-clades. However, the only acquisition of a mobile element by the main branch is GI33A, a Lambda-like bacteriophage, which also did not contain any obvious cargo genes. We conclude that our genomes do not provide support for any association of a mobile element with increased virulence, or the ability to cause an outbreak. Instead, the extensive diversity of the accessory genome might largely reflect the fact that the mobile elements are behaving like ‘selfish DNA’, whose spread from bacterial strain to strain benefits the mobile element, but not necessarily the host bacterium. In that event, we would expect only a limited correlation between the presence or absence of mobile elements and the genealogy defined by core SNPs. The presence of genomic islands and accessory genes in the 72 new genomes were strongly correlated (coefficient of determination *R* ^2^ = 0.46 between matrices of genetic similarities in pair-wise comparisons). Similar comparisons of each of these matrices with a matrix of the core nucleotide variants that differed between pairs of genomes also yielded a significant Mantel correlation. However, as expected, the coefficients of determination were lower than between accessory genes and genomic islands (Figure 8A–8C).

Our results suggest that any Agona isolate is potentially able to cause an outbreak, and are compatible with the absence of Darwinian selection within Agona for ability to cause disease or outbreaks. We cannot exclude the possibility that the ability of Agona to cause outbreaks is exclusively associated with the main branch, and was acquired together with its branch-specific properties, including its 31 SNPs, two short indels and/or 14 recombinational tracts. We note, however, that the two other branches have persisted through to modern days despite being only rarely isolated, and also cause sporadic disease in humans.

### Epidemiological Markers of Outbreak-Specific Genetic Diversification

All of the outbreaks investigated here were caused by highly uniform clades, with no more than seven non-repetitive, non-mobile SNPs that distinguished between any pair of isolates from a single outbreak, with one exception. Despite this homogeneity in the core genome, epidemiologists were partially successful in recognizing single source outbreaks on the basis of PFGE. This partial success reflected differences in the accessory genome, as shown by the strong correlation between genetic similarities according to PFGE and either accessory genes (Figure 8D) or genomic islands (Figure 8E). In contrast, PFGE correlated only poorly with SNPs (Figure 8F).

Clade A consists of two sub-clades: A1 caused a small human outbreak in Ireland in 2005, and A2 was concurrently isolated from Irish pigs and poultry (Figure 1). Sub-clades A1 and A2 differ dramatically in PFGE pattern, and A2 possesses the XB.66 PFGE pattern that was typical of the Irish ready to eat food outbreak three years later in 2008, except for two isolates which differed by one band (AgoX50). The 2008 outbreak was associated with sub-clades D1, which was isolated from the waste water in the food factory, and D2, which was isolated from food, infected humans, swine and water. D2 was associated with the XB.66 pattern but D1 was associated with the AgoX67 pattern, which differs by two bands. We have successfully reconstructed the PFGE patterns from the genomic assemblies, with only very few bands that cannot be accounted for (Figure 1). In PFGE pattern AgoX3 (seven isolates in sub-clade A1), the unexplained bands might represent a genomic inversion (Figure S8). Such genomic rearrangements might also account for the three other PFGE patterns with discrepant bands but we cannot exclude the possibility that these discrepancies represent rare local misassemblies with SOAPdenovo [59].

Our reconstructions of the genomic basis of PFGE patterns showed that the two-band difference between D1 and D2 reflects the deletion of P2-like phage GI25A (Figure S1, Dataset S1). On the other hand, the XB.66 PFGE pattern common to isolates of the A2 and D2 sub-clades reflects the fact that they both contain Fels-2-like bacteriophage GI21A. Although indistinguishable by PFGE pattern, the A and D clusters are genealogically unrelated, differing by 39 of the 722 core, non-recombinant SNPs that were variable among the 70 genomes within the main branch. However, due to their identical PFGE patterns, one sub-clade A2 strain was incorrectly assigned to the 2008 outbreak because it was isolated in that year. Sub-clades A1 and A2 differ by only three SNPs. These sub-clades differ by five PFGE bands, two of which are accounted for by GI24A which is related to ICESe4 in serovar Hadar. Similarly, sub-clades D1 and D2 differ by only two SNPs but two PFGE bands (GI25A; see above).

Clade C encompassed three isolates from a German outbreak in 2002–2003, which was associated with aniseed from Turkey [28], and which were clustered together by PFGE despite multiple visual differences. Indeed, one of these, pattern AgoX91, was originally considered the same as AgoX89 and AgoX90 in branch E. After examination of the genetic distance matrix, reevaluation revealed that their PFGE patterns are distinct. These PFGE similarities between genetically distinct clades reflect their possession of similar Fels-2-like bacteriophages, whereas the differences in band patterns between XB.10 and AgoX91 within clade C are due to different variants of the Fels-2-like bacteriophage plus the effects of a deletion of the SPI-1 genomic island in the AgoX91 strain 39.O.03. SPI-1 is generally considered essential for virulence of *S. enterica*, but this might not be true for human infections because other isolates from food-borne gastroenteritis of humans also lack SPI-1 [60]. The SPI-1 deletion extended through the neighboring *mutS* gene. Bacteria lacking the MutS protein are mutators, and strain 39.O.03 differs from the other clade C isolates by 14 SNPs, but it is clearly within the same genealogical clade (Figure 1).

Clade B provides still a different perspective on outbreaks because it includes sub-clades B1, which was associated with toasted oat cereal in the USA in 1998, and B2, which was associated with contaminated cereal from the same food plant 10 years later [24], [26]. Clade B1 includes the Agona reference strain (SL483, here 25.H.03), which was isolated from sporadic disease in the USA in 2003, and only differs from the former outbreak isolates by one SNP and three additional ISN16 elements. B1 and B2 differ by six core SNPs [61]. These isolates have the same PFGE profiles (XB.24a), which are just distinguishable by expert practitioners from very similar patterns in unrelated strains called XB.24b and XB.24c.

**Figure 9:**
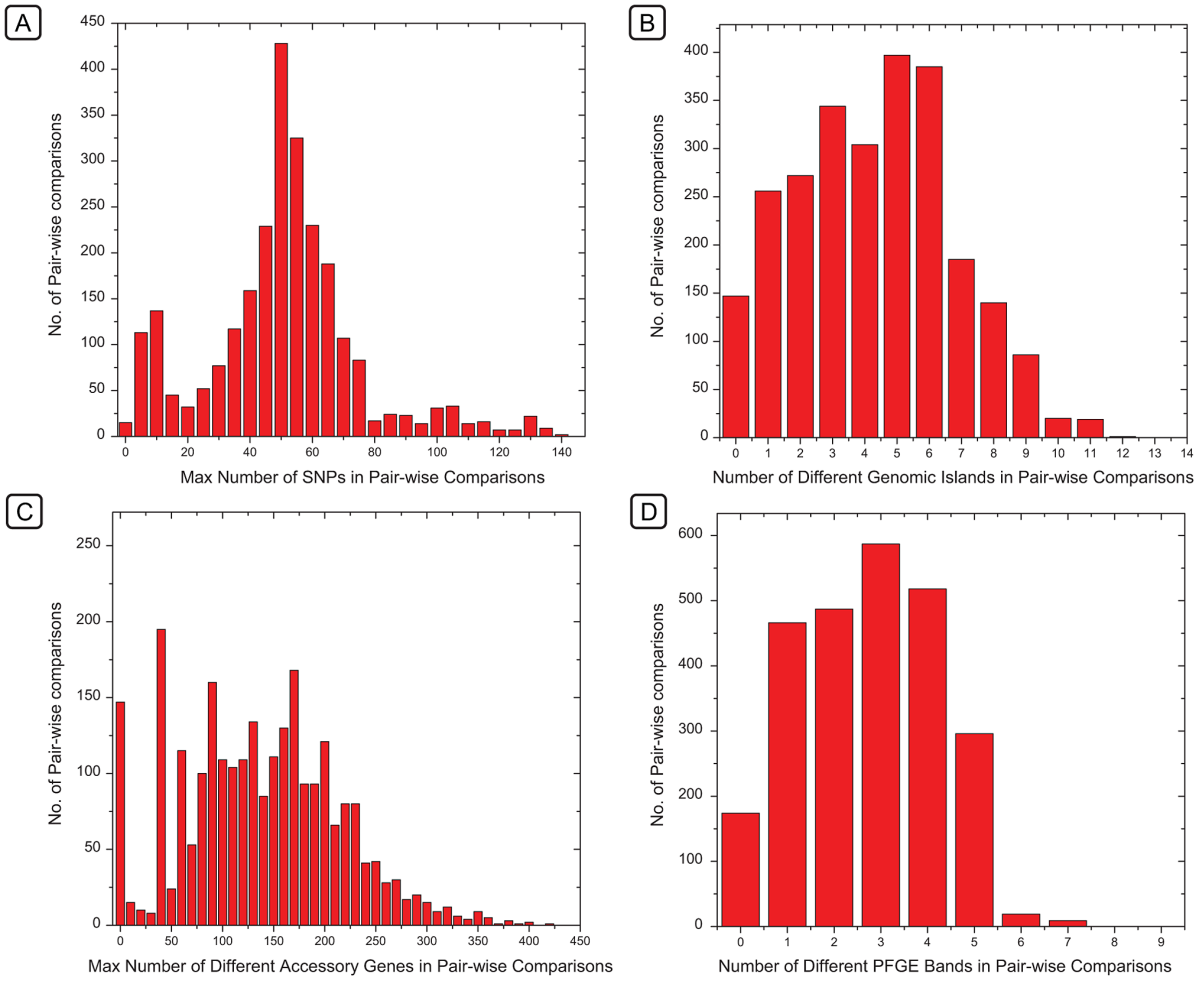
Histograms of the frequencies of numbers of differences between pairs of genomes in SNPs, genomic islands, accessory genes, or PFGE bands. Histograms of the frequencies of numbers of differences between pairs of genomes in SNPs (A), genomic islands (B), accessory genes (C), or PFGE bands (D). The X axes of histograms A and C indicate the maximal number of differences within the indicated ranges in order to ensure that the first column only includes identical pairs (maximum = 0). These frequency distributions correspond to the same data which were used for comparisons in Figure 8, except that those referred to genetic similarity and the data here refer to the converse, which are differences.

Our observations indicate that the criteria of identical or closely related PFGE patterns coupled with epidemiological linkage of isolates can indeed help to identify single-source outbreaks [5]. This partial success is surprising. The number of PFGE bands that differed between individual isolates formed a continuum, as did the number of different genomic islands (Figure 9B, 9D). Based on these observations, a clear cut-off between closely related and more distant bacteria, such as has been proposed for outbreaks by Tenover *et al.* [4], should be difficult to define on the basis of either PFGE or genomic islands. In contrast, the number of different SNPs (Figure 9A) or accessory genes (Figure 9C) showed a clear peak of identical (accessory genes) or nearly identical (SNPs) strains within clades or sub-clades, clearly separated from other isolates with greater diversity.

Our detailed comparison of the PFGE patterns and the accessory genomes showed that different PFGE patterns reflected myriad changes in mobile elements of the accessory genome, particularly Fels-2-like phages, which frequently changed their genomic locations, or were lost. The longevity of these genomic associations is variable in different clades, and some PFGE patterns distinguish between almost identical bacteria while others conflate unrelated bacteria. In particular, the relationships between isolates from the 2008 Irish outbreak and those from the waste water in the contaminating plant were uncertain [1] because they differed by two PFGE bands. Similarly, the relationships between 2005 isolates from domesticated animals in Ireland and the 2008 outbreak were also uncertain because their PFGE patterns were identical. Similar uncertainties existed for the epidemiological relationships between isolates from cereal grains from 1998 and 2008 in the USA, which possessed identical PFGE patterns [61]. We conclude that such stochastic changes in PFGE patterns are not the optimal basis for decisions on public health interventions, which can also have important economic consequences. Instead, we show that genomic sequencing provides a solid basis to augment and replace the existing bacterial typing systems that are used for public health decisions. This potential can be implemented as soon as current procedures for the generation and evaluation of genomic sequencing have been adapted to the real-time needs of diagnostic laboratories.

## Materials and Methods

### Strain Selection

The properties and sources of the 73 Agona strains are listed in Dataset S2. We tested 15 strains with the XB.66 PFGE profile from Irish poultry and pigs in 2005 and from the multi-country outbreak in 2008. Eleven other strains from the waste water at the source food plant had the AgoX67 PFGE profile, which differs from XB.66a by two bands. To identify additional strains with increasing levels of PFGE pattern dissimilarity to XB.66, a database of PFGE patterns was assembled based on 309 isolates from Ireland (National *Salmonella* Reference Laboratory, Galway and The Centre for Food Safety, Univ. College Dublin); and 148 isolates from Scotland (Scottish *Salmonella*, *Shigella* and *Clostridium difficile* Reference Laboratory, Glasgow) for a total of 457 isolates. These PFGE patterns were compared by Dice similarity, with a band matching tolerance of 1% and a pattern matching optimization parameter of 1%, using Bionumerics v. 6.5 (Applied-Maths, Sint Martens Latem, Belgium). Eighteen isolates were selected that represented various levels of Dice similarity to XB.66 ranging from 0.28–0.92, with priority given to PFGE patterns that were common in the database. Fourteen other strains represent other outbreaks (USA 1998 & 2008 [4 strains, XB.24a], Germany 2002–2003 [3 strains, AgoX91 & XB.42] and Ireland 2005 [7 strains, AgoX3]). Finally, 14 strains were chosen to represent diverse geographical sources (including Austria, Denmark, Germany, Taiwan and the United Arab Emirates) and dates, including the first typed Agona strain from 1952 and two isolates from England in 1972. We also included the published genomic sequence of the reference strain SL483, which was isolated from a sporadic human infection in the USA in 2003 [62].

### Genomic DNA Preparation and Sequencing

DNA was prepared from 5 ml overnight cultures with the Jetflex Genomic DNA Purification Kit (Genomed, Germany) according to the manufacturer's instructions. Whole genome sequencing was performed on 68 strains using an Illumina GAIIx on 250 bp paired-end libraries in 8-fold multiplexes (University of Toronto, Canada) and on four other strains on a HiSeq 2000 in 7-fold multiplexes (Institut Pasteur, Paris, France). Details for each genomic assembly are summarized in Dataset S2, including the accession codes at the EMBL sequence read archive (Study ERP001848 with Samples ERS180311-382, Experiments ERX155006-077 and Runs ERR178860-931) and EMBL genomic assemblies at www.ebi.ac.uk/ena (PRJEB1064-1135).

### *De Novo* Assembly

Reads were assembled *de novo* using SOAPdenovo [59] with optimal Kmer and minimal coverage parameters. Intra-scaffold gaps were filled using GapCloser v1.10 (SOAP package). The average values for 72 genomes were: read coverage depth, 173-fold; number of scaffolds, 171; and total length, 4,782,841 (Dataset S2). After completion of assemblies, SOAPaligner v2.21 [63] was used to remap the reads to the assembled scaffolds for validation of the quality of each base that was called in the assembly. Three SOAPaligner parameters were varied depending on read length: 34 bp (-g: 17; -v: 8; -s:17), 74 bp (-g: 30; -v: 15; -s:30) or 101 bp (-g: 40; -v: 25; -s:40). We filtered sites with a quality score of <20 or a read coverage of <10.

### Reconstructions of Genomic Islands and Gap Filling

A short-read genomic assembly consists of a set of N scaffolds each containing one or more contigs, which we designated as S_i_ (1<i< = N). The sequences at the ends of each scaffold S_i_ are designated S_iL_ and S_iR_,. The paired reads from the ends of inserts (approximately 250 bp long in our sequences) can be used to close gaps, even when no reads overlap the internal, unsequenced contents of inserts. Mapping the read-pairs to the ends of all scaffolds was used to identify short gaps of 300 bp that had not been closed by our assemblies (Figure S9A). Mapping resulted in sets of connections between pairs of scaffold ends. In cases where multiple sets of connections were found, we accepted all connections with at least two read hits and which did not conflict with the reference genome SL483.

Reconstructing GIs faces additional difficulties due to repetitive DNA which is common in GIs. Some gaps in GIs were closed as in Figure S9A. In other cases involving short tandem repeats, BLASTn searches of unmapped reads with one end mapping to a scaffold allowed gap closure as in Figure S9B. For other paired-end reads with multiple, alternative connections we used the following sequential strategies.

We resolved some gaps in which reads had mapped to the ends of tiny contigs (<300 bp), which had not been assembled into scaffolds, as well as to the ends of scaffolds (Figure S9C–S9D). These tiny contigs provided missing sequence information with gaps on each end which were used for GI reconstruction, except that it was not possible to reconstruct their internal order when too many tiny contigs were involved (Figure S9D). The same strategy was used when an internal contig also contained internal connections due to larger tandem repeats (Figure S9F). In other cases involving repetitive sequences (Figure S9E), multiple scaffold ends were linked to a common contig. The order of such scaffolds was determined by mapping their internal contents to SL483. For the two cases where these scaffolds did not align with the reference genome (GI1D and GI4 in genome 20.H.06) we were unable to assign one third of the CDSs of these two seroconverting phages directly from an analysis of paired-read connection. In this case, the GI contents were assigned on the basis of the homologies of those CDSs with other, similar seroconverting phages in other genomes.

Repetitive DNA can also result in both ends of scaffolds being connected to the same scaffold due to genomic inversions in part of the DNA used for sequencing (Figure S9G), direct repeats spanning scaffolds (Figure S9H), or inverted repeats (Figure S9I). For genomic inversions, we did not annotate the direction of the inverted segment within the GI. For direct repeats, we eliminated circular connections to provide a linear, shortened repeat region containing as few as one repeat unit. However, only few contigs among the Agona genomes contained >2 tandem repeats within a single GI. These tandem repeats were short and did not possess any *XbaI* restriction sites. For inverted repeats, we left the chromosomal order of the circular element unresolved.

### Core Genome Assignment

The assembled genomes were aligned to the reference strain SL483 using the nucmer module within MUMmer [64] with the ‘–mum’ parameter. Regions that were absent in any of the 73 genomes were removed. Where multiple regions aligned to a common position in the reference, we retained the aligned region with the highest quality. This filtering process was carried out with the delta-filter utility in the MUMmer package [64], and subsequently refined using a perl script. Three approaches were used to identify repeat regions, which were subsequently also removed from the core genome. These included: 1) multiple segments of >50 bps dispersed throughout SL483 with a BLASTn hit of >94% identity when SL483 was blasted against itself; 2) VNTRs identified by Tandem Repeat Finder 4.04 [65]; 3) CRISPR1 and CRISPR2 regions. After removing repeat regions and mobile elements, the residual core genome comprised 4.2 Mb, which was retained for further analysis (Dataset S10).

Recombinant segments within the aligned non-repetitive, non-mobile 4.2 Mb core genome were identified with ClonalFrame [34] in the three most divergent Agona genomes (strains 71.E.05, 72.A.52, 73.H.09). To this end, two independent runs of ClonalFrame were performed, each consisting of 20,000 iterations of which the first 10,000 were discarded as the MCMC burn-in. Convergence between the two runs was confirmed based on good mixing between the runs and consistent estimates of global parameters, location of recombination events and inferred clonal genealogies. Recombinant segments were defined as regions in which each polymorphic nucleotide had a probability of recombination >0.3. Using cut-offs of greater than 0.3 yielded comparable results whereas lower cut-off values resulted in variable calls of recombinant fragments. Two additional recombinant segments within the main branch in nodes N05-N06 and N17-18.H.07 were identified by visual inspection of clustered SNPs positions for all genomes.

### SNP Calls and Phylogenetic Inference

The core genome contained 849 non-recombinational SNPs, from which a Maximum Parsimony (MP) phylogeny was inferred. A perl script was used to identify 3 homoplastic sites which were subsequently removed and a final MP tree was derived in Mega v4 [66]. The ancestral root was determined by comparison to other genomic sequences from the distantly related serovars Enteritidis (P125109), Paratyphi B (SPB7), Typhimurium (T000240) and Newport (SL254).

### Root-to-Tip Distances

Based on the 846 parsimonious SNPs, a maximum likelihood tree was inferred using PhyML [67] applying the TVM+G model, which had the best-fit of sequence evolution according to the Akaike Information Criterion (AIC) using Multiphyl [68]. Linear regression of root-to-tip distances against date of isolation was performed using Path-O-Gen (http://tree.bio.ed.ac.uk/software/pathogen/).

### Bayesian Estimates of Age and Population Fluctuation

The population history of the Agona dataset, including age estimates and mutation rates, were inferred using Beast v1.7.1 [36]. Alternative clock models and tree priors that were based on a strict clock rate and on a relaxed, uncorrelated lognormal clock rate were tested under the assumption of a constant coalescent population. We also tested the Gaussian Markov random fields (GMRF) option to allow for population size fluctuations with the relaxed clock model. Bayes factor (BF) tests were used to evaluate differences in the outcome under the alternative models, which indicated that our data best fit the relaxed molecular clock with a constant population size (Table S3). The BF was ln 2.1 stronger for this model than the GMRF model, but such small BF values correspond to only weak support [69]. We performed further analyses using the GMRF model despite its weaker BF support because a fluctuating population size is more appropriate considering the known demographic history of Agona. The population history of the Agona dataset was inferred using the Bayesian GMRF skyride plot [36]. A Maximum clade credibility tree was constructed using TreeAnnotator v1.5.3 illustrating rate variations across the phylogeny (Figure S3). The date of the TMRCA for all Agona was estimated along with the dates of individual outbreaks (Table S3). All analyses were run with chain lengths of 50 million and model parameter values were sampled every 5,000 generations. Model performance was assessed in Tracer v1.5, which indicated that all effective sample size values were >300.

### SNP Densities

Pairwise comparisons were used to investigate the average SNP densities within Agona, and between Agona and other serovars. For each comparison, the genomes of two strains were aligned using the nucmer module within MUMmer and filtering was carried out with MUMmer's delta-filter option and a Perl script. All-versus-all pairwise comparisons were performed for the 73 Agona genomes (Figure S4). Agona SL483 was also compared in pair-wise fashion to each of 12 genomes from serovars Choleraesuis, Dublin, Enteriditis, Gallinarum, Heidelberg, Newport, Paratyphi A, Paratyphi B, Paratyphi C, Schwarzengrund, Typhi and Typhimurium.

### Pan-Genome Gene Prediction

CDSs from the reference genome SL483 were compiled into a training-set to create an interpolated Markov model in Glimmer 3.02 [70]. This model was implemented to predict *ab initio* CDSs in the 72 assemblies. The predicted CDSs were then combined with CDSs in the reference genome into a dataset of all putative CDSs in Agona. That dataset was then used to query (BLASTn) all 73 assembled Agona genomes. All blast hits with an E-value<1e-5 and ≥80% identity were retrieved as High Scoring Matches (HSMs). For each query gene, multiple HSMs in close proximity on the genome were merged into a single HSM, after which only HSMs that covered ≥60% of the query sequence were retained as homologous regions of the query CDS. Some HSMs of query CDSs are interrupted by gaps containing regions not covered by reads in the assemblies, resulting in fragmented regions that span the start and end points of contigs. In such cases, a homologous region was called when ≥40% of the total length of the query CDS was covered by the HSMs located at the ends of contigs. Subsequently, homologous groups were formed based on the homologous regions for each query CDS that overlapped by *≥*40% of their length. For homologous groups containing more than one CDS from the reference, the longest SL483 CDS was chosen as a representative. If a homologous CDS was lacking from SL483, the longest homologous CDS in the CDS dataset was chosen to represent the group. All other CDSs were removed as redundant, resulting in a pan-genome of CDSs.

### Pan-Genome Annotation

CDSs in the pan-genome were translated and used to search the non-redundant UniRef100 dataset in UniProtKB [71] and the eggNOG 3.0 database [72] using BLASTp. Putative gene annotations and functions were assigned to CDSs using the criteria of E-value<1e-05 and identity ≥30%. HMM based gene prediction methods such as Glimmer have been reported to have a tendency to predict small unreliable CDSs. Therefore, CDSs<100 amino acids lacking a clear match or a clear functional designation were removed from the pan-genome, leaving a total of 6,102 pan-genome CDSs for genomic island reconstruction.

### Identification of IS Elements

Protein sequences for all 6,102 pan-genome CDSs were aligned to the ISfinder database [73] using the online BLASTp tool. CDS hits that passed the criteria of E-value<1e-05, ≥60% identity and ≥80% length coverage were assigned as putative transposases. Transposases with multiple hits within 1 Kb in the assemblies to a common IS element were scored as a single IS, resulting in the identification of 41 distinct IS elements. Designations of ISs were based on BLASTn searches of the nucleic acid sequences of all 41 IS elements against the ISfinder database (BLASTn), and IS names and families were assigned based on the best hit.

### Terminal Ends of IS Elements

The reads for each genome assembly were aligned to the nucleic acid sequences of the 41 IS elements using SOAPaligner. We retrieved unpaired reads, consisting of read-pairs from which only one read mapped to the IS sequences. These were then mapped to the assemblies (SOAPaligner) to identify potential conjunction sites for IS elements as clusters of unpaired reads in which at least two reads mapped within 100 bps. These potential conjunction sites were scored as confirmed if unpaired reads in both directions were included in the clusters. Copy numbers were calculated as the number of conjunction sites found per assembly for each IS element.

### Identification of Small Indels

Small insertions and deletions (indels) (≤11 bps) in the core genome were identified by using SSAHA2 [74] with an insert size between 50–400 to map reads to the assemblies. We used SSAHA2 rather than SOAP Aligner because SSAHA2 uses local alignments for these purposes, which is more appropriate for identifying small indels than SOAP, which requires at least one of the paired-end reads to be fully mapped before it recognizes an indel. The analysis of the Samtools output was performed with Dindel [75], which was one of the two most accurate among a variety of down-stream analyzers of such data [35] and is superior to the other analyzer when coverage is low.

### CRISPR Regions

Sequencing reads were aligned to the sequences of two CRISPR regions that had been previously described in Agona [54] using SOAPaligner. The resulting ‘pair’ output file was converted to SAM format using SOAP2SAM.pl (SOAPaligner package), and to BAM format using the ‘view’ option in Samtools. The ‘pileup’ option within Samtools was used to show read coverage at each site. Sites without read coverage represent spacer deletions. We also searched for spacer insertions by examining the BAM file for locations where only parts of a read were mapped, but none were found

### Virulence Factors

CDSs in the accessory genome were blasted against the known *Salmonella* virulence factors in the VFDB (Virulence Factors of Pathogenic Bacteria database) [76] using both BLASTp and tBLASTn. We identified only one cluster of virulence factors in genomic island 22 (SPI-1), as well as the *gogB* gene in genomic island 6 (Gifsy-1).

In an independent approach, we searched specifically for type three secretion systems (TTSS), which are thought to be important for the ability of bacterial pathogens to inject “effector proteins” into host cells. We therefore blasted >300 proven or predicted TTSS effectors (Dataset 1 in [77]) against the pan genome using BLASTp and tBLASTn. However, all hits to TTSS effectors overlapped with the virulence factors identified by the first approach.

### *In Silico* PFGE

Pseudo-genomes were created for each of the 72 assemblies by modifying a copy of the reference genome of SL483 *via* the introduction or deletion of all mobile elements that were identified within the assembly, and by updating the SNPs. *XbaI* restriction sites were then localized within each pseudogenome. Two restriction sites in the core genome (SL483 positions 3,683,315 and 4,538,898) were excluded because they are subject to Dam methylation, which prevents cleavage [78]. Observed PFGE band sizes between 30 and 800 kb were calculated from PFGE patterns using Bionumerics 6.5 and a pattern mapping optimization parameter of 1.0% and a band matching tolerance parameter of 0.7% with Dice similarity. The number of different bands between pairs of strains were calculated from the similarity matrix that is output by Bionumerics after normalizing according to the Dice correlation for the average number of bands in each pair of strains. The calculated cleaved fragments for each pseudo-genome were ordered by length and compared to the band sizes from the PFGE patterns. The similarity matrix was used for Figure 8D–8F and the pair-wise differences in number of bands were used for Figure 9D.

### Statistical Tests of Neutrality

For Figure 7A, we clustered all 73 genomes into 4 outbreak and 28 non-outbreak sub-clades that differed by at least 20 core SNPs. We calculated the relative frequency in outbreak and non-outbreak clades for each CDS within the accessory genome using the Fisher exact test of significance. Finally, a Bonferroni correction was applied to each test to account for the fact that 1,582 separate tests had been performed, after which no CDS yielded a value lower than 0.05. For Figure 7B and Figure S5, we applied the methods described elsewhere by Jombard [58] using an R script which he kindly supplied. We confirmed the results in Figure S7 with independently coded scripts in perl.

## Supporting Information

Dataset S1 Genomic islands and plasmids in all 73 isolates of serovar Agona.
(XLSX)

Dataset S2 Properties of genomic sequences.
(XLSX)

Dataset S3 Presence/absence matrix of PFGE bands for 72 new genomes.
(XLSX)

Dataset S4 Details of mutational SNPs and short indels in the core genome.
(XLSX)

Dataset S5 Details of the SNPs and indels in the core genome within recombination regions.
(XLSX)

Dataset S6 CDSs that contains SNPs due to recombinations in the non-repetitive, non-mobile core genome of serovar Agona.
(XLSX)

Dataset S7 Accessory genes.
(XLSX)

Dataset S8 Details of CRISPR variants.
(XLSX)

Dataset S9 Potential functional CDSs in genomic islands in all 73 isolates of serovar Agona.
(XLSX)

Dataset S10 Details of the core genome.
(XLSX)

Figure S1 Genealogy of 73 Agona genomes based on non-recombinant, non-mobile SNPs in the core genome versus insertions and deletions associated with mobile elements. The genealogy reflects a maximum parsimony tree based on 846 core SNPs (as in Figure 1), but drawn in radial fashion (Mega) for convenience. The tips of the branches include strain ID numbers, as in Figure 1 and Dataset S2. Node, clade and branch designations are also according to Figure 1 and Dataset S2. Mobile elements that are inserted are shown with solid lines, and dashed lines indicate deletions (Key). Their GI codes are according to Dataset S1.
(PDF)

Figure S2 Distribution of the size of the accessory genome in 73 serovar Agona strains. Strains are ordered by diminishing size of their accessory genomes.
(PDF)

Figure S3 Genealogy of 73 Agona genomes based on SNPs in the non-recombinant, non-mobile core genome versus insertions (red) and deletions (black) (Dataset S4). Other details are as in Figure S1 except for the designations of the different types of insertions/deletions.
(PDF)

Figure S4 Root-to-tip genetic distances to the MRCA of non-recombinant, non-mobile SNPs in the core genome *versus* dates of sampling. Each data point represents a distinct genome. A linear regression is indicated by the straight line, whose correlation coefficient was 0.3, as indicated. The mean TMRCA according to a Beast v1.7.1 analysis with a relaxed GMRF model is depicted immediately above the X-axis, together with the 95% confidence intervals of that estimated mean.
(PDF)

Figure S5 Genealogy of 73 Agona genomes based on non-recombinant, non-mobile SNPs in the core genome versus insertions (red) and deletions (black) of CRISPR spacers. Other details are as in Figure S1, except that the designations indicate spacer designations (Dataset S8).
(PDF)

Figure S6 Insertions (solid lines) and deletions (dashed lines) of potential cargo CDSs in ICE/IMEs (red boxes), other genomic islands (red triangles) and plasmids (red circles). These genomic changes are mapped on a SNP genealogy of 73 Agona genomes based on non-recombinant, non-repetitive, non-mobile core SNPs. Other details are as in Figure S1 except that the GI codes of the mobile elements are according to Dataset S1.
(PDF)

Figure S7 Identification of non-repetitive, non-mobile core CDSs with unexpected frequencies of non-synonymous mutations. A) Parametric and nonparametric χ^2^ goodness-of-fit test of the expected number of non-synonymous mutations per gene. The observed χ^2^ (red arrow) is distinct from both the expectations according to a parametric distribution (black line) and according to a simulated distribution (blue histograms). B).Percentage contribution of each gene to the deviation from the expected number of non-synonymous mutations, ordered by decreasing contribution. The main part of the figure shows the 20 genes with the highest contributions, and the entire distribution is shown in the inset. Gene lengths are indicated by colors (Key). The only significant outlier is separate from other genes by a black arrow.
(PDF)

Figure S8 Correspondence and discrepancies between *in silico* predictions and observed XbaI-digested PFGE patterns. (A) Observed *versus* predicted PFGE patterns. Dashed black lines indicate bands in pattern AgoX3 (sub-clade A1) whose presence/absence cannot be explained by *in silico* prediction. All other bands in AgoX3 and all bands in AgoX67 and XB.66 correspond between the predicted and observed patterns (B). Circular pseudo-genomes based on insertions or deletions of mobile elements into SL438 that show the genomic positions of XbaI-digested fragments. The dashed arrows in AgoX3 represent a potential genomic rearrangement that could account for the discrepancies between the predictions and the observed patterns. Grey shaded boxes outside the circles indicate bacteriophages, whose sizes are also indicated.
(PDF)

Figure S9 Decision tree used to fill gaps and reconstruct genomic islands. Panels A-I illustrate all possible types of gaps in assemblies. Blue lines indicate scaffolds, and curved lines with arrows indicate connections between the scaffolds. In panels A and B, both ends of the read pairs are represented as red and green lines, with dashed lines showing connections between paired-ends. In panels A, H and I, decisions regarding connections between scaffolds are indicated by a vertical arrow. (A) Gaps due to low sequencing coverage. (B) Gaps that are interrupted by tandem repeats with short repetitive units (<100 bp). (C–D) Two examples of connections between tiny scaffolds (<300 bp). (E) Gaps that are interrupted by repeat regions (S_5_). (F) Gaps that are interrupted by tandem repeats with larger repetitive units (>100 bp, S_2_). (G) Regions involving inversions in both directions (S_2_). (H) Fragments (S_2_) that are surrounded by direct repeats (S_4_). (I) Fragments (S_2_) that are surrounded by inverted repeats (S_4_). Greater details on these procedures are provided in Materials and Methods under the heading “Reconstructions of Genomic Islands and Gap Filling”.
(PDF)

Table S1 Summary of sources of strains.
(DOCX)

Table S2 Numbers of features of the Agona accessory genome.
(DOCX)

Table S3 Homoplastic SNPs and short indels in the core Agona genome.
(DOCX)

Table S4 Beast dating estimates of the age of Agona according to strict and relaxed mutation rates.
(DOCX)

Table S5 Comparison of clock rates from different experiments.
(DOCX)

Table S6 Recombination regions identified by ClonalFrame.
(DOCX)

Table S7 Invariable IS elements in the core genome.
(DOCX)

Table S8 Variable IS insertions in the core genome.
(DOCX)

Table S9 IS elements in genomic islands or plasmids.
(DOCX)
