# Supplementary figures and images for: Neutral Genomic Microevolution of a Recently Emerged Pathogen, Salmonella enterica Serovar Agona
Source: PLoS Genet. 2013 Apr 18;9(4):e1003471. doi: 10.1371/journal.pgen.1003471 (PMC3630104; doi:10.1371/journal.pgen.1003471)

# Key

|            |                  |              |
|------------|------------------|--------------|
| ●          | Node designation |              |
| ◇          | Cluster/Branch   |              |
| <b>Ins</b> | <b>Del</b>       | <b>Event</b> |
| ⊠          | ⊠                | Phage/ICE    |
| ⊙          | ⊙                | Plasmid      |
| ⊡          | ⊡                | Genomic      |

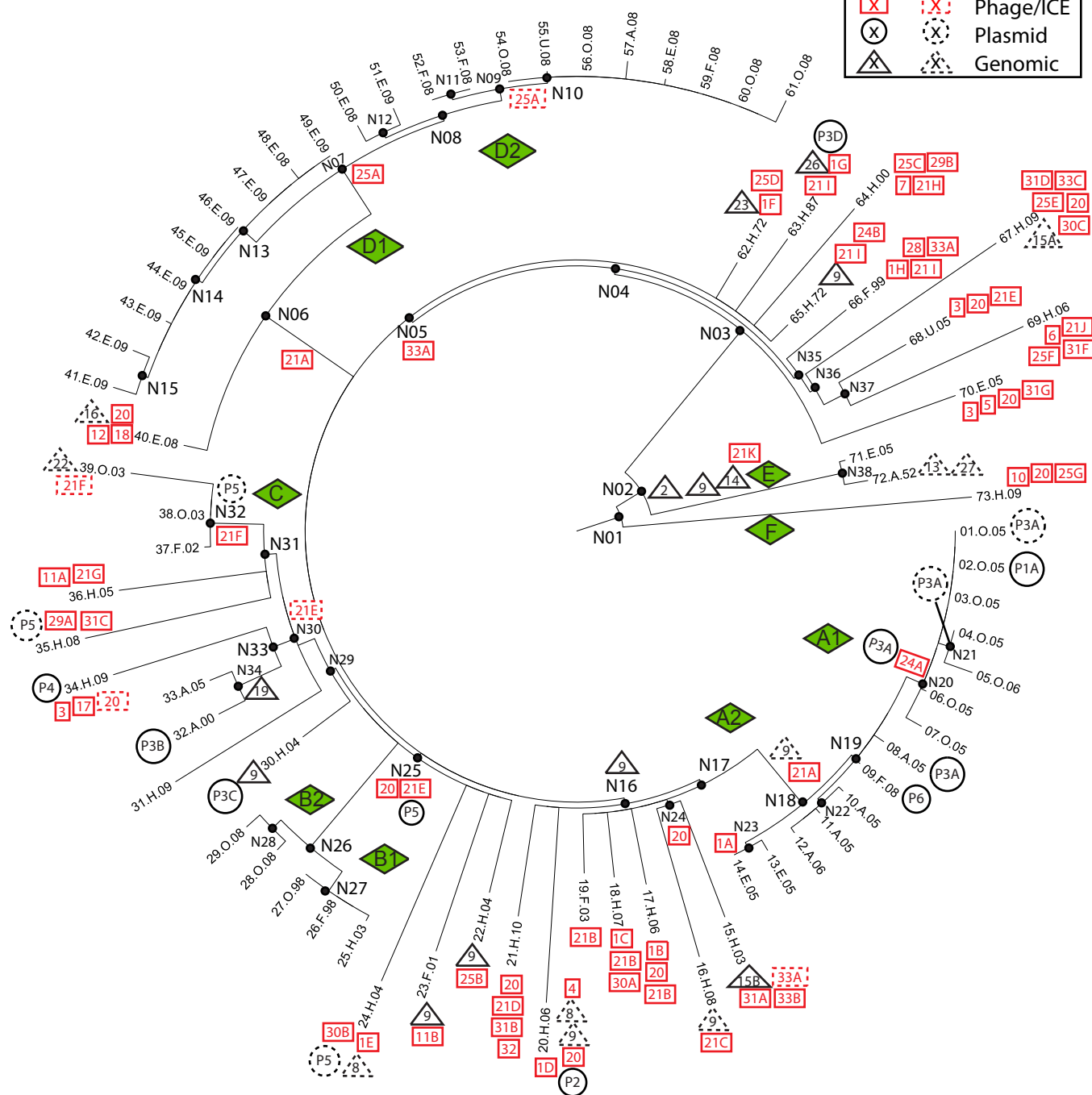

Supplement: Figure S1 — Genealogy of 73 Agona genomes based on non-recombinant, non-mobile SNPs in the core genome versus insertions and deletions associated with mobile elements. The genealogy reflects a maximum parsimony tree based on 846 core SNPs (as in Figure 1), but drawn in radial fashion (Mega) for convenience. The tips of the branches include strain ID numbers, as in Figure 1 and Dataset S2. Node, clade and branch designations are also according to Figure 1 and Dataset S2. Mobile elements that are inserted are shown with solid lines, and dashed lines indicate deletions (Key). Their GI codes are according to Dataset S1. (PDF) [file pgen.1003471.s011.pdf]

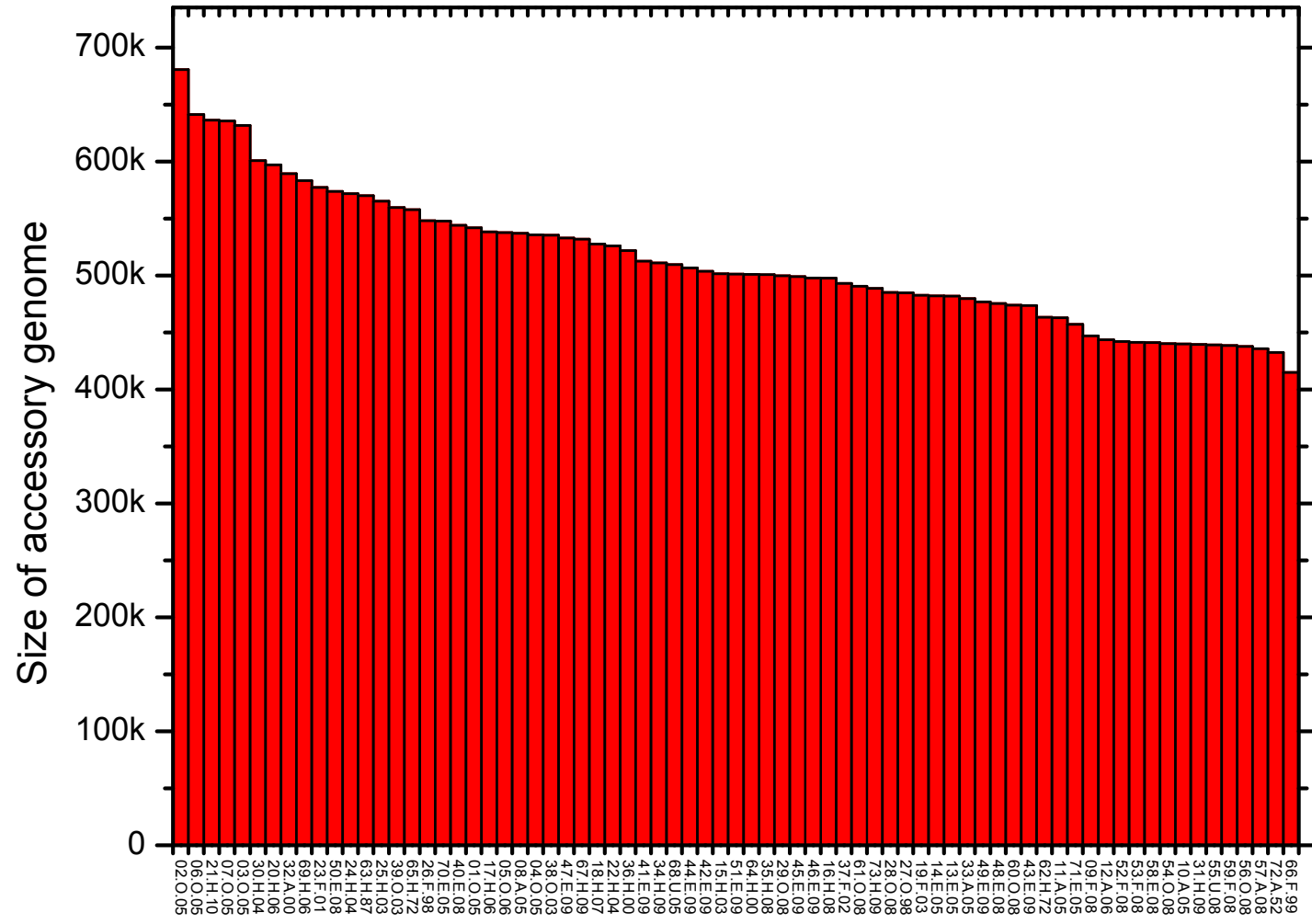

Supplement: Figure S2 — Distribution of the size of the accessory genome in 73 serovar Agona strains. Strains are ordered by diminishing size of their accessory genomes. (PDF) [file pgen.1003471.s012.pdf]

# Key

|           |                |                |            |
|-----------|----------------|----------------|------------|
|           | Cluster/Branch | ●              | Node       |
|           | Frameshift     | Non-frameshift | Non-coding |
| Insertion |                |                |            |
| Deletion  |                |                |            |

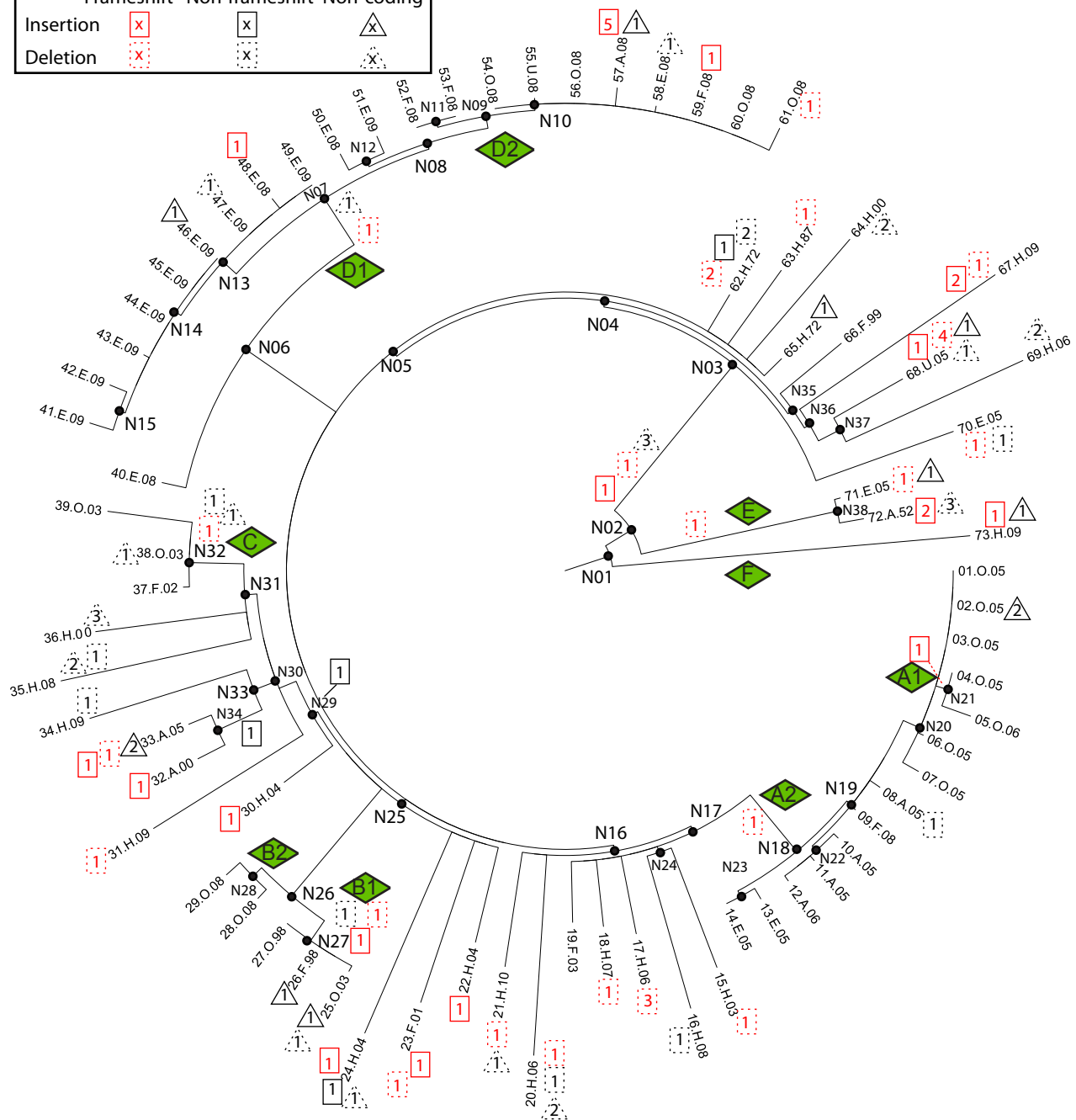

Supplement: Figure S3 — Genealogy of 73 Agona genomes based on SNPs in the non-recombinant, non-mobile core genome versus insertions (red) and deletions (black) (Dataset S4). Other details are as in Figure S1 except for the designations of the different types of insertions/deletions. (PDF) [file pgen.1003471.s013.pdf]

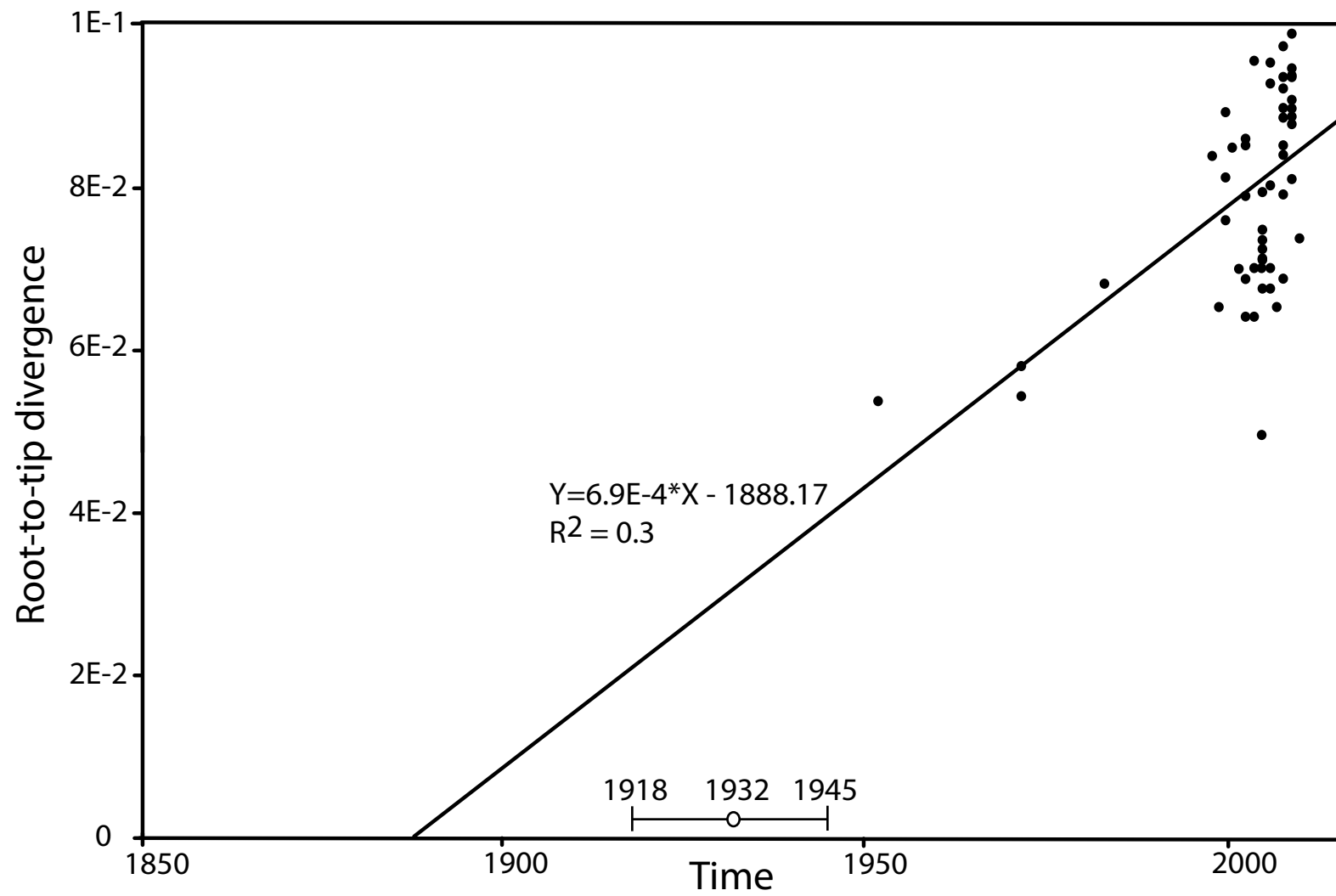

Supplement: Figure S4 — Root-to-tip genetic distances to the MRCA of non-recombinant, non-mobile SNPs in the core genome versus dates of sampling. Each data point represents a distinct genome. A linear regression is indicated by the straight line, whose correlation coefficient was 0.3, as indicated. The mean TMRCA according to a Beast v1.7.1 analysis with a relaxed GMRF model is depicted immediately above the X-axis, together with the 95% confidence intervals of that estimated mean. (PDF) [file pgen.1003471.s014.pdf]

# Key

|            |                         |
|------------|-------------------------|
| ●          | Node designation        |
| ◆          | Cluster/Branch          |
| <i>Ins</i> | <i>Del</i> <b>Event</b> |
| ⊠          | ⊠ ICE/IME               |
| ⊗          | ⊗ Plasmid               |
| ⊗          | ⊗ Genomic               |

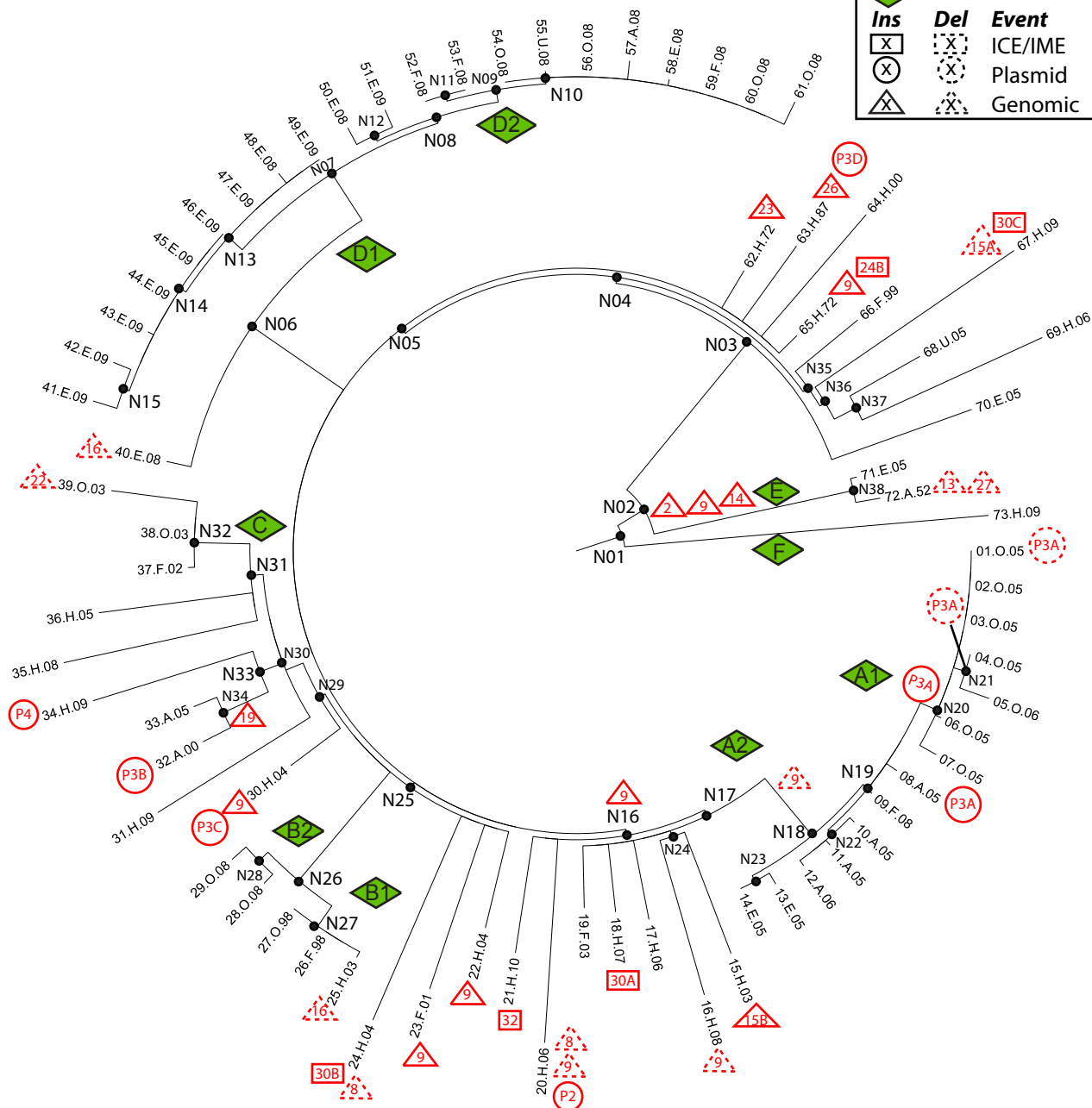

Supplement: Figure S6 — Insertions (solid lines) and deletions (dashed lines) of potential cargo CDSs in ICE/IMEs (red boxes), other genomic islands (red triangles) and plasmids (red circles). These genomic changes are mapped on a SNP genealogy of 73 Agona genomes based on non-recombinant, non-repetitive, non-mobile core SNPs. Other details are as in Figure S1 except that the GI codes of the mobile elements are according to Dataset S1. (PDF) [file pgen.1003471.s016.pdf]

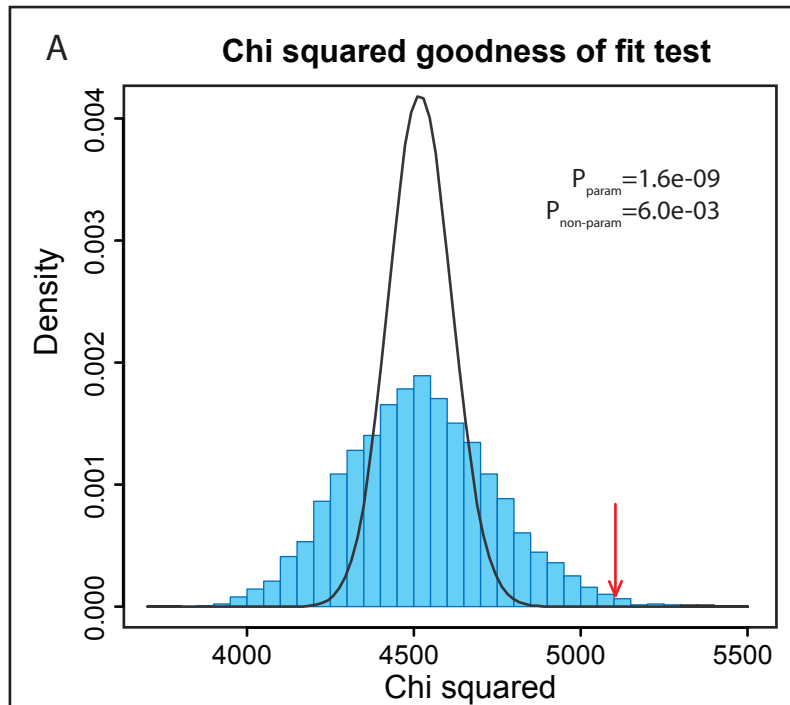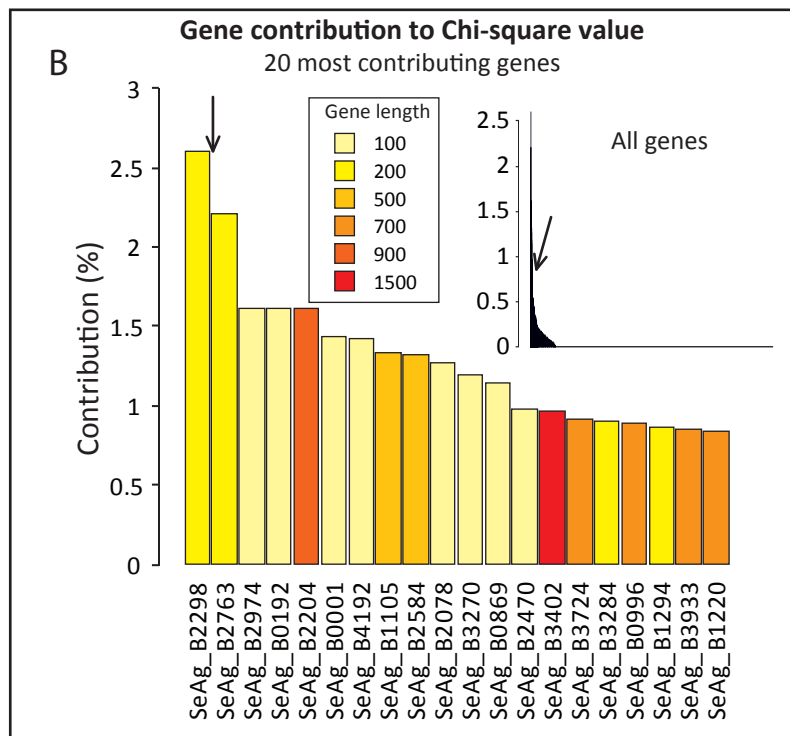

Supplement: Figure S7 — Identification of non-repetitive, non-mobile core CDSs with unexpected frequencies of non-synonymous mutations. A) Parametric and nonparametric χ2 goodness-of-fit test of the expected number of non-synonymous mutations per gene. The observed χ2 (red arrow) is distinct from both the expectations according to a parametric distribution (black line) and according to a simulated distribution (blue histograms). B).Percentage contribution of each gene to the deviation from the expected number of non-synonymous mutations, ordered by decreasing contribution. The main part of the figure shows the 20 genes with the highest contributions, and the entire distribution is shown in the inset. Gene lengths are indicated by colors (Key). The only significant outlier is separate from other genes by a black arrow. (PDF) [file pgen.1003471.s017.pdf]

A

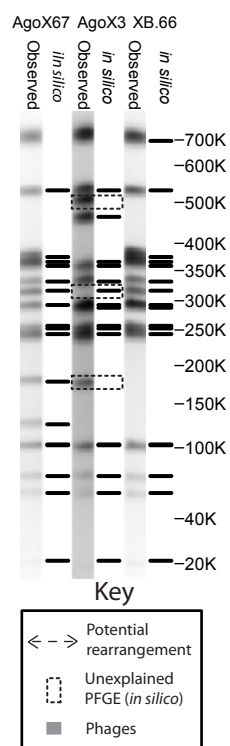

B

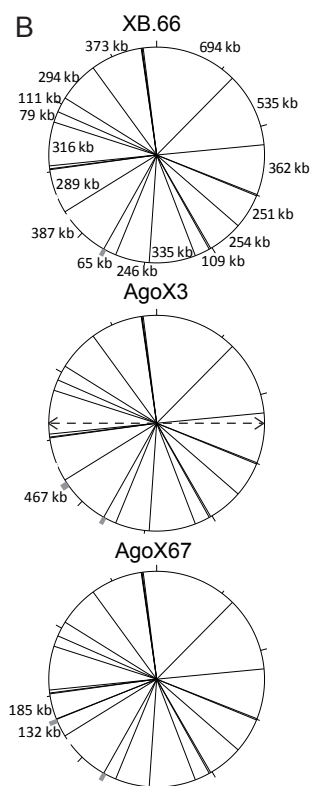

Supplement: Figure S8 — Correspondence and discrepancies between in silico predictions and observed XbaI-digested PFGE patterns. (A) Observed versus predicted PFGE patterns. Dashed black lines indicate bands in pattern AgoX3 (sub-clade A1) whose presence/absence cannot be explained by in silico prediction. All other bands in AgoX3 and all bands in AgoX67 and XB.66 correspond between the predicted and observed patterns (B). Circular pseudo-genomes based on insertions or deletions of mobile elements into SL438 that show the genomic positions of XbaI-digested fragments. The dashed arrows in AgoX3 represent a potential genomic rearrangement that could account for the discrepancies between the predictions and the observed patterns. Grey shaded boxes outside the circles indicate bacteriophages, whose sizes are also indicated. (PDF) [file pgen.1003471.s018.pdf]

# Reconstruction of genomic islands and gap filling

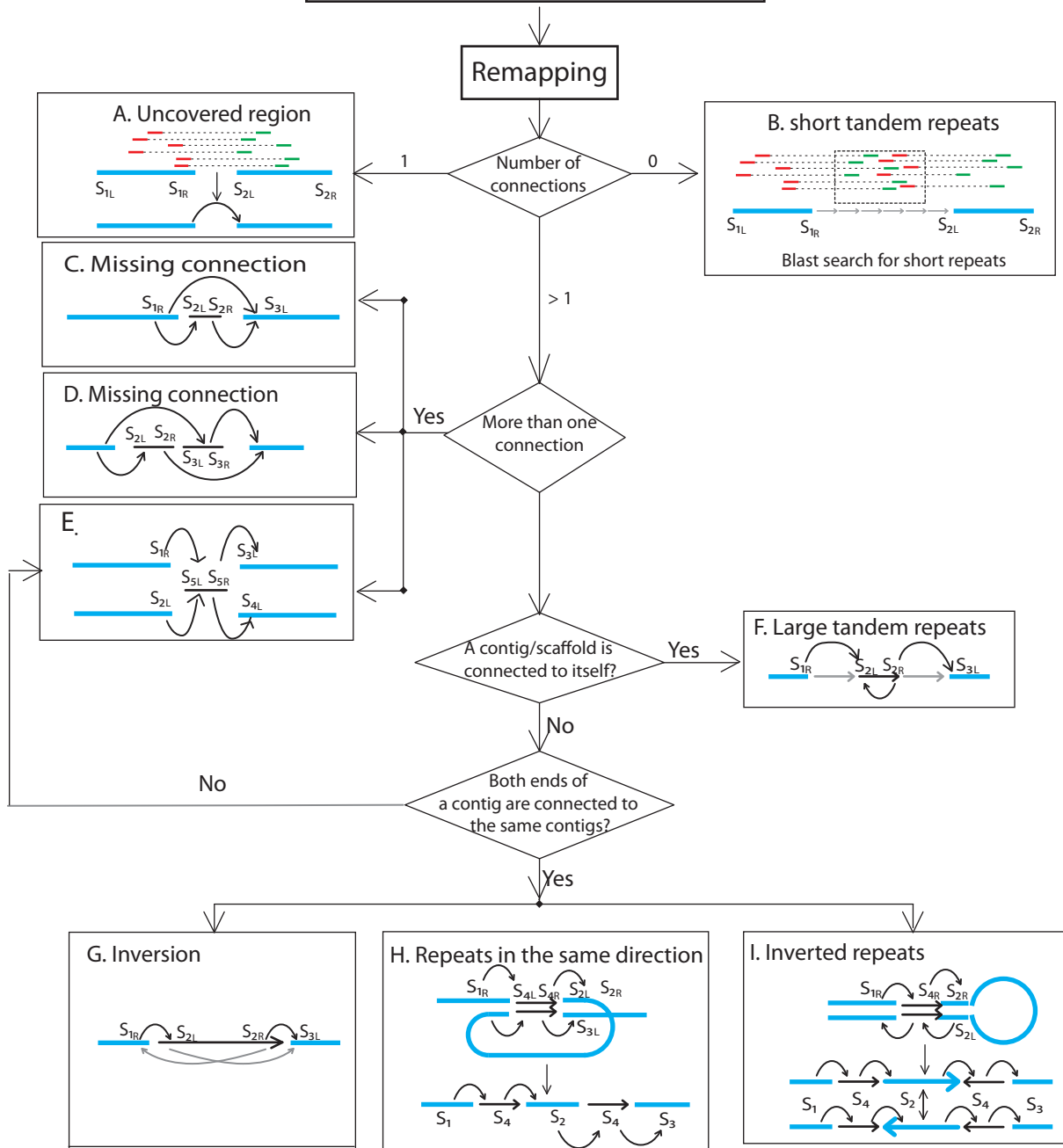

Supplement: Figure S9 — Decision tree used to fill gaps and reconstruct genomic islands. Panels A-I illustrate all possible types of gaps in assemblies. Blue lines indicate scaffolds, and curved lines with arrows indicate connections between the scaffolds. In panels A and B, both ends of the read pairs are represented as red and green lines, with dashed lines showing connections between paired-ends. In panels A, H and I, decisions regarding connections between scaffolds are indicated by a vertical arrow. (A) Gaps due to low sequencing coverage. (B) Gaps that are interrupted by tandem repeats with short repetitive units (<100 bp). (C–D) Two examples of connections between tiny scaffolds (<300 bp). (E) Gaps that are interrupted by repeat regions (S5). (F) Gaps that are interrupted by tandem repeats with larger repetitive units (>100 bp, S2). (G) Regions involving inversions in both directions (S2). (H) Fragments (S2) that are surrounded by direct repeats (S4). (I) Fragments (S2) that are surrounded by inverted repeats (S4). Greater details on these procedures are provided in Materials and Methods under the heading “Reconstructions of Genomic Islands and Gap Filling”. (PDF) [file pgen.1003471.s019.pdf]
