# Supplementary material for: Neutral Genomic Microevolution of a Recently Emerged Pathogen, Salmonella enterica Serovar Agona
Source: PLoS Genet. 2013 Apr 18;9(4):e1003471. doi: 10.1371/journal.pgen.1003471 (PMC3630104; doi:10.1371/journal.pgen.1003471)
Supplement: Figure S5 — Genealogy of 73 Agona genomes based on non-recombinant, non-mobile SNPs in the core genome versus insertions (red) and deletions (black) of CRISPR spacers. Other details are as in Figure S1, except that the designations indicate spacer designations (Dataset S8). (PDF) [file pgen.1003471.s015.pdf]

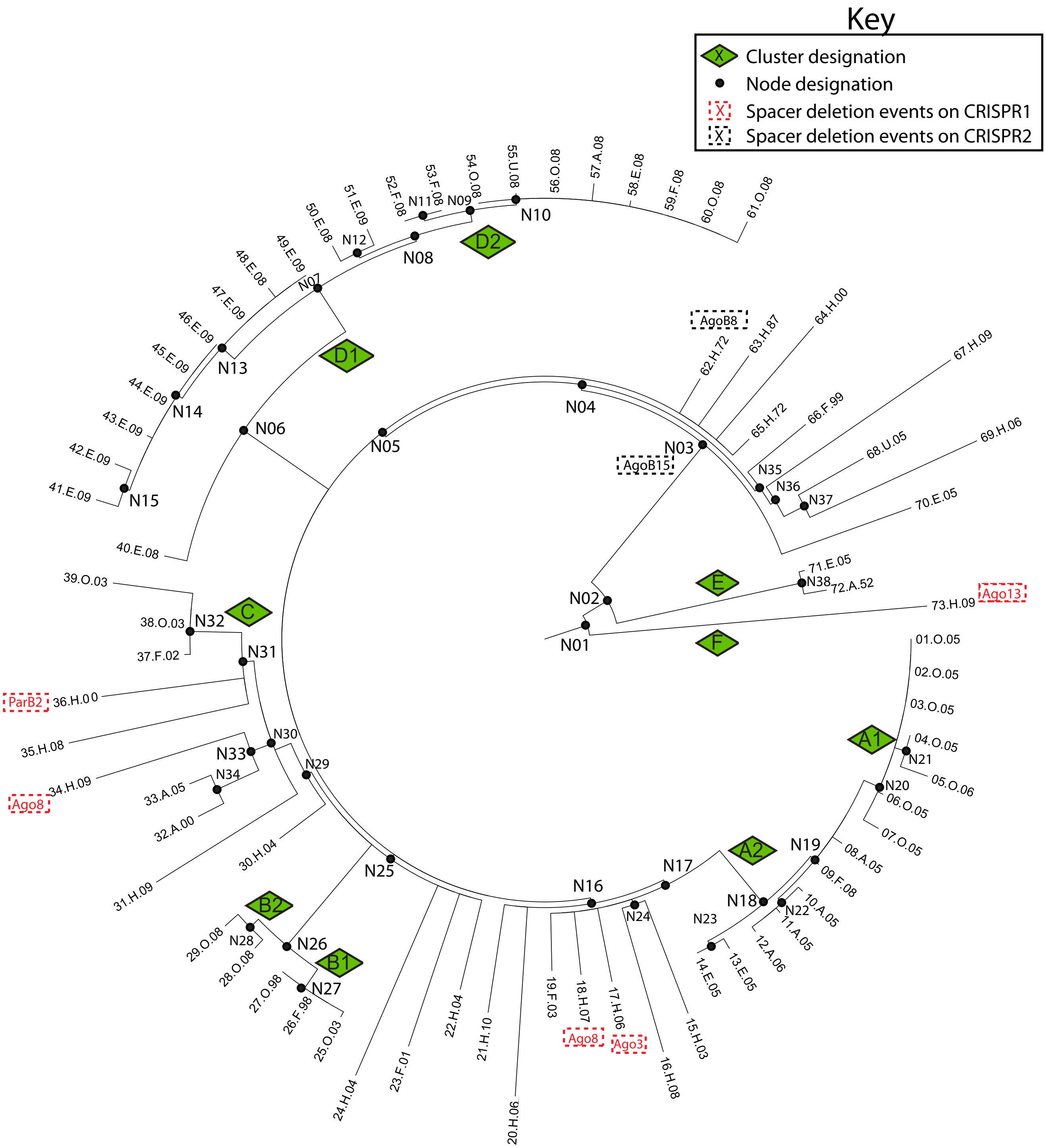
Key
Cluster designation
Node designation
Spacer deletion events on CRISPR1
Spacer deletion events on CRISPR2
A1
A2
B1
B2
C
D1
D2
E
F
N01
N02
N03
N04
N05
N06
N07
N08
N09
N10
N11
N12
N13
N14
N15
N16
N17
N18
N19
N20
N21
N22
N23
N24
N25
N26
N27
N28
N29
N30
N31
N32
N33
N34
N35
N36
N37
N38
01.O.05
02.O.05
03.O.05
04.O.05
05.O.06
06.O.05
07.O.05
08.A.05
09.F.08
10.A.05
11.A.05
12.A.06
13.E.05
14.E.05
15.H.03
16.H.08
17.H.06
18.H.07
19.F.03
20.H.06
21.H.10
22.H.04
23.F.01
24.H.04
25.O.03
26.F.98
27.O.98
28.O.08
29.O.08
30.H.04
31.H.09
32.A.00
33.A.05
34.H.09
35.H.08
36.H.00
37.F.02
38.O.03
39.O.03
40.E.08
41.E.09
42.E.09
43.E.09
44.E.09
45.E.09
46.E.09
47.E.09
48.E.08
49.E.09
50.E.08
51.E.09
52.F.08
53.F.08
54.O.08
55.U.08
56.O.08
57.A.08
58.E.08
59.F.08
60.O.08
61.O.08
62.H.72
63.H.87
64.H.00
65.H.72
66.F.99
67.H.09
68.U.05
69.H.06
70.E.05
71.E.05
72.A.52
73.H.09
AgoB8
AgoB15
Ago13
ParB2
Ago8
Ago8
Ago3
