# Supplementary material for: Neutral Genomic Microevolution of a Recently Emerged Pathogen, Salmonella enterica Serovar Agona
Source: PLoS Genet. 2013 Apr 18;9(4):e1003471. doi: 10.1371/journal.pgen.1003471 (PMC3630104; doi:10.1371/journal.pgen.1003471)
Supplement: Table S1 — Summary of sources of strains. (DOCX) [file pgen.1003471.s020.docx]

**Table S1**. Summary of sources of strains.

| **Source** | | **Number** |
| --- | --- | --- |
| **Epidemiological** | **Geographical** |  |
| **Clusters related to outbreaks** | |  |
| A1 (human 2005) | Ireland | 7 |
| A2 (swine/poultry 2005) | Ireland | 7 |
| B1 (Food/Human 1998) | USA | 3 |
| B2 (Human 2008) | USA | 2 |
| C (Food/Human 2002/2003) | Germany | 3 |
| D1 (Factory wastewater 2008-2009) | Ireland | 11 |
| D2 (Food outbreak 2008) | Ireland, Scotland | 10 |
| **Geographical Origin** | |  |
| Ireland |  | 37 |
| Scotland |  | 14 |
| Germany |  | 7 |
| Austria, Denmark, England |  | 5 |
| Taiwan |  | 2 |
| UAE |  | 2 |
| Ghana |  | 1 |
| USA |  | 5 |
| **Human** | | 37 |
| **Environment** | | 17 |
| **Animal** | | 8 |
| **Food** | | 9 |

**Note**: Clusters related to outbreaks are subdivided genetically as according
to Fig. 1 and Dataset S2
