# Supplementary material for: Neutral Genomic Microevolution of a Recently Emerged Pathogen, Salmonella enterica Serovar Agona
Source: PLoS Genet. 2013 Apr 18;9(4):e1003471. doi: 10.1371/journal.pgen.1003471 (PMC3630104; doi:10.1371/journal.pgen.1003471)
Supplement: Table S2 — Numbers of features of the Agona accessory genome. (DOCX) [file pgen.1003471.s021.docx]

**Table S2.** Numbers of features of the Agona accessory genome.

|  | CDS | Mobile  Element | Insertions | Deletions |
| --- | --- | --- | --- | --- |
| Accessory Genome: | 1,582 | 150 | 148 | 30 |
| Insertion Elements | 59 | 41 | 54 | 8 |
| Genomic islands | 94 | 12 | 9 | 12 |
| Bacteriophages | 889 | 51 | 69 | 5 |
| ICEs | 242 | 3 | 3 | 0 |
| IMEs | 43 | 3 | 3 | 0 |
| Plasmids | 255 | 10 | 11 | 5 |

**Note:** The accessory genome CDSs span a total of ~1,273 Kb, after excluding intergenic regions. ICE – Integrative Conjugative Element; IME – Integrative Mobile Element.
