# Supplementary material for: Neutral Genomic Microevolution of a Recently Emerged Pathogen, Salmonella enterica Serovar Agona
Source: PLoS Genet. 2013 Apr 18;9(4):e1003471. doi: 10.1371/journal.pgen.1003471 (PMC3630104; doi:10.1371/journal.pgen.1003471)
Supplement: Table S3 — Homoplastic SNPs and short indels in the core Agona genome. (DOCX) [file pgen.1003471.s022.docx]

**Table S3.** Homoplastic SNPs and short indels in the core Agona genome.

| **Mutation ID** | **Position in SL483** | **Lineage** | **Locus tag** | **Gene name** | **NT Change** | **Codon Change** | **AA Change** | **Type*** | **Product** |
| --- | --- | --- | --- | --- | --- | --- | --- | --- | --- |
| s245A | 1206755 | N25-23.F.01 | SeAg_B1220 | *-* | a->c | ttt->gtt | Phe->Val | NS | hypothetical protein |
| s245B | 1206755 | N31-36.H.00 | SeAg_B1220 | *-* | a->c | ttt->gtt | Phe->Val | NS | hypothetical protein |
| s246A | 1206756 | N25-23.F.01 | SeAg_B1220 | *-* | g->a | cac->cat | His->His | S | hypothetical protein |
| s246B | 1206756 | N31-36.H.00 | SeAg_B1220 | *-* | g->a | cac->cat | His->His | S | hypothetical protein |
| s612A | 3046104 | N01-73.H.09 | SeAg_B3121 | *fucI* | t->a | cca->cct | Pro->Pro | S | L-fucose isomerase |
| s612B | 3046104 | N02-N38 | SeAg_B3121 | *fucI* | t->a | cca->cct | Pro->Pro | S | L-fucose isomerase |
| s631A | 3011557 | N02-N03 | - | *-* | G->. | - | - | DEL | - |
| s631B | 3011557 | N16-20.H.06 | - | *-* | G->. | - | - | DEL | - |

**Note:** These mutations are also listed in Dataset S2.
^*^S - synonomous; NS - non-synonomous; DEL - deletion; INS – insertion.
Lineage is according to Fig. S1. The mutation IDs of the four homoplastic SNPs are s245, s246, s612 and s631.
