# Supplementary material for: Neutral Genomic Microevolution of a Recently Emerged Pathogen, Salmonella enterica Serovar Agona
Source: PLoS Genet. 2013 Apr 18;9(4):e1003471. doi: 10.1371/journal.pgen.1003471 (PMC3630104; doi:10.1371/journal.pgen.1003471)
Supplement: Table S4 — Beast dating estimates of the age of Agona according to strict and relaxed mutation rates. (DOCX) [file pgen.1003471.s023.docx]

**Table S4.** Beast dating estimates of the age of Agona according to strict and relaxed mutation rates.

|  | **Relaxed Clock** | | | | **Strict Clock** | |
| --- | --- | --- | --- | --- | --- | --- |
|  | **GMRF** | | **Constant Population Size** | | **Constant Population Size** | |
| **Node (cluster)** | **Mean MRCA** | **95% Confidence Interval** | **Mean MRCA** | **95% Confidence Interval** | **Mean MRCA** | **95% Confidence Interval** |
| MRCA (node N01) | 1932 | 1918-1945 | 1799 | 1618-1928 | 1839 | 1765-1894 |
| N03 | 1949 | 1937-1960 | 1942 | 1910-1966 | 1927 | 1892-1954 |
| N07 (D1) | 1997 | 1987-2004 | 1997 | 1989-2003 | 1994 | 1986-2001 |
| N09 (D2) | 2004 | 2000-2006 | 2003 | 2001-2007 | 2001 | 1999-2006 |
| N18 (A2) | 1993 | 1982-2001 | 1997 | 1991-2002 | 1993 | 1984-1999 |
| N20 (A1) | 2001 | 1997-2004 | 2002 | 1999-2004 | 1999 | 1995-2003 |
| N26 (MRCA: B1-B2) | 1988 | 1977-1996 | 1991 | 1984-1997 | 1990 | 1982-1996 |
| N32 (C) | 1993 | 1983-2001 | 1998 | 1993-2002 | 1984 | 1972-1995 |

**Note:** ln Bayes factor scores: Relaxed GMRF *versus* Relaxed Constant: -2.1;
Relaxed GMRF *versus* Strict Constant: 69.6; Relaxed Constant *versus* Strict Constant: 71.7
