# Supplementary material for: Neutral Genomic Microevolution of a Recently Emerged Pathogen, Salmonella enterica Serovar Agona
Source: PLoS Genet. 2013 Apr 18;9(4):e1003471. doi: 10.1371/journal.pgen.1003471 (PMC3630104; doi:10.1371/journal.pgen.1003471)
Supplement: Table S5 — Comparison of clock rates from different experiments. (DOCX) [file pgen.1003471.s024.docx]

**Table S5.** Comparison of clock rates from different experiments

| **Taxon** | **Citation** | **Clock rate (95% CI)** |
| --- | --- | --- |
| *Helicobacter pylori* experimental infection | 1 | 2.5 × 10^-5^ (0.5 - 6.5 × 10^-5^) |
| *Staphylococcus aureus* (ST239) | 2 | 3.3 × 10^-6^ (2.5 - 4.0 × 10^-6^) |
| *Streptococcus pneumoniae* (ST81) | 3 | 1.6 × 10^-6^ (1.3 - 1.9 × 10^-6^) |
| *Helicobacter pylori* Sequential isolates (MLST) | 4 | 1.4 × 10^-6^ (0.9 – 1.9 × 10^-6^) |
| *Vibrio cholerae* (7^th^ pandemic) | 5 | 8.3 × 10^-7^ |
| *Burkholderia dolosa* | 6 | 3.0 × 10^-7^ |
| *Helicobacter pylori* over 50,000 years (MLST) | 4 | 2.6 × 10^-7^(1.8 - 3.6 × 10^-7^) |
| *Buchnera aphidicola* | 7 | 2.2 × 10^-7^ |
| *S. enterica* serovar Agona Relaxed Constant | Current study | 1.4 × 10^-7^(8.6 × 10 ^-8^ - 2.1 ×10^-7^) |
| *S. enterica* serovar Agona Strict Constant | Current study | 1.3 × 10^-7^ (1.1 - 1.7 ×10^-7^) |
| *Mycobacterium tuberculosis* | 8 | 8.8 × 10^-8^ - 1.3 × 10^-7^ |
| *S. enterica* serovar Agona Relaxed GMRF | Current study | 9.3 × 10^-8^ (5.7 × 10^-8^ - 1.3 ×10^-7^) |
| *Clostridium difficile* | 9 | 2.5 × 10^-9^ - 1.5 × 10^-8^ |
| *Yersinia pestis* | 10 | 8.6 × 10^-9^ |

1. L. Kennemann *et al.*, *Proc. Natl. Acad. Sci. (USA)* **108**, 5033 (2011).

2. R. Harris *et al.*, *Science* **327**, 469 (2010).

3. N. J. Croucher *et al.*, *Science* **331**, 430 (2011).

4. G. Morelli *et al.*, *PLoS Genet* **6**, e1001036 (2010).

5. A. Mutreja *et al.*, *Nature* **477**, 462 (2011).

6. T. D. Lieberman *et al.*, *Nature Genet.* **43**, 1275 (2011).

7. N. A. Moran, H. J. McLaughlin, R. Sorek, *Science* **323**, 379 (2009).

8. C. B. Ford *et al.*, *Nature Genet.* **43**, 482 (2011).

9. M. He *et al.*, *Proc. Natl. Acad. Sci. (USA)* **107**, 7527 (2010).

10. G. Morelli *et al.*, *Nature Genet.* **42**, 1140 (2010).
