# Supplementary material for: Neutral Genomic Microevolution of a Recently Emerged Pathogen, Salmonella enterica Serovar Agona
Source: PLoS Genet. 2013 Apr 18;9(4):e1003471. doi: 10.1371/journal.pgen.1003471 (PMC3630104; doi:10.1371/journal.pgen.1003471)
Supplement: Table S6 — Recombination regions identified by ClonalFrame. (DOCX) [file pgen.1003471.s025.docx]

**Table S6.** Recombination regions identified by ClonalFrame

|  |  |  |  |  | **No. of SNPs*** | | | |  |  | **No. of Genes** |
| --- | --- | --- | --- | --- | --- | --- | --- | --- | --- | --- | --- |
| **REC_ID** | **Lineage** | **Start** | **End** | **Length** | **NS** | **S** | **NC** | **STOP** | **INS** | **DEL** |  |
| **REC001** | N02-73.H.09 | 161389 | 179675 | 18190 | 26 | 95 | 17 | - | - | 1 | 21 |
| **REC002** | N02-73.H.09 | 1489560 | 1500825 | 10914 | 17 | 77 | 14 | 1 | - | - | 9 |
| **REC003** | N02-73.H.09 | 1552676 | 1553539 | 760 | - | 5 | 4 | - | 1 | - | 1 |
| **REC004** | N02-73.H.09 | 1558216 | 1566711 | 8019 | 3 | 32 | 22 | - | - | - | 6 |
| **REC005** | N02-73.H.09 | 1568952 | 1579234 | 9971 | 26 | 60 | 14 | - | 1 | - | 6 |
| **REC006** | N02-73.H.09 | 1676169 | 1676791 | 623 | - | 4 | - | - | - | - | 2 |
| **REC007** | N02-73.H.09 | 2384782 | 2384805 | 24 | 3 | 3 | - | - | - | - | 1 |
| **REC008** | N02-73.H.09 | 2398035 | 2399132 | 1001 | - | 4 | - | - | - | - | 2 |
| **REC009** | N02-73.H.09 | 2883458 | 2903752 | 19925 | 45 | 121 | 16 | - | - | 1 | 22 |
| **REC010** | N02-73.H.09 | 3027146 | 3029167 | 2016 | - | 9 | - | - | - | - | 2 |
| **REC011** | N02-73.H.09 | 3039718 | 3047566 | 5397 | - | 22 | - | - | - | - | 6 |
| **REC012** | N02-73.H.09 | 3221553 | 3228808 | 7229 | 8 | 34 | 1 | - | - | - | 9 |
| **REC013** | N02-73.H.09 | 3489400 | 3499603 | 10138 | 11 | 55 | 9 | - | - | - | 12 |
| **REC014** | N02-73.H.09 | 3527167 | 3529351 | 2117 | 6 | 27 | 7 | - | 1 | - | 4 |
| **REC015** | N02-N38 | 682 | 12060 | 11263 | 7 | 44 | 5 | 1 | - | - | 11 |
| **REC016** | N02-N38 | 113295 | 114003 | 622 | - | - | 5 | - | - | - | 1 |
| **REC017** | N02-N38 | 197882 | 199676 | 1633 | 1 | 11 | 1 | - | - | - | 4 |
| **REC018** | N02-N38 | 360798 | 430150 | 66108 | 111 | 409 | 95 | - | 3 | 2 | 60 |
| **REC019** | N02-N38 | 603063 | 633196 | 29028 | 60 | 187 | 28 | - | 0 | 2 | 25 |
| **REC020** | N02-N38 | 754539 | 755262 | 722 | 1 | 3 | 7 | - | 1 | 1 | 1 |
| **REC021** | N02-N38 | 1458270 | 1473313 | 13992 | 39 | 97 | 14 | - | - | - | 12 |
| **REC022** | N02-N38 | 1711656 | 1732022 | 19701 | 72 | 141 | 18 | - | 1 | - | 26 |
| **REC023** | N02-N38 | 1733727 | 1736975 | 3181 | 3 | 15 | 1 | - | - | - | 3 |
| **REC024** | N02-N38 | 2979471 | 2987706 | 8138 | 5 | 50 | 2 | - | - | - | 11 |
| **REC025** | N02-N38 | 3020488 | 3020955 | 468 | - | 6 | - | - | - | - | 1 |
| **REC026** | N02-N38 | 3024396 | 3040171 | 15693 | 12 | 84 | 6 | - | - | - | 13 |
| **REC027** | N02-N38 | 3043434 | 3050212 | 6445 | 5 | 39 | 2 | - | - | - | 7 |
| **REC028** | N02-N38 | 3971265 | 3973612 | 2348 | 1 | 12 | 1 | - | 1 | - | 4 |
| **REC029** | N02-N03 | 138759 | 149286 | 10321 | 9 | 37 | 4 | - | - | - | 12 |
| **REC030** | N02-N03 | 219558 | 246076 | 24612 | 48 | 182 | 13 | - | 1 | - | 24 |
| **REC031** | N02-N03 | 907841 | 921079 | 12729 | 16 | 73 | 10 | - | - | - | 15 |
| **REC032** | N02-N03 | 1948617 | 1950899 | 2183 | 1 | 14 | - | - | - | - | 4 |
| **REC033** | N02-N03 | 2203364 | 2207634 | 3675 | 12 | 34 | - | - | - | 1 | 6 |
| **REC034** | N02-N03 | 2557482 | 2564230 | 5760 | 1 | 19 | 31 | - | - | - | 6 |
| **REC035** | N02-N03 | 3027188 | 3029425 | 2231 | 3 | 18 | 1 | - | - | - | 3 |
| **REC036** | N02-N03 | 3039640 | 3040180 | 541 | - | 6 | - | - | - | - | 1 |
| **REC037** | N02-N03 | 3042614 | 3048171 | 5263 | 5 | 13 | 2 | - | - | - | 6 |
| **REC038** | N02-N03 | 3439659 | 3445881 | 5994 | 3 | 55 | 10 | - | - | - | 9 |
| **REC039** | N02-N03 | 4124166 | 4128183 | 3969 | 5 | 35 | 1 | - | - | - | 4 |
| **REC040** | N02-N03 | 4257013 | 4263663 | 6582 | 5 | 45 | 5 | - | - | - | 6 |
| **REC041** | N05-N06 | 1116044 | 1116079 | 36 | 8 | 2 | - | - | - | - | 1 |
| **REC042** | N17-18.H.07 | 599574 | 599763 | 189 | 3 | 36 | - | - | - | - | 1 |

**Note:** *S - synonymous; NS - non-synonymous; NC - non-coding; STOP - nonsense mutation; INS – insertion; DEL – deletion. Lineages are named according to Dataset S2. Start and end positions are relative to the SL483 genome. Length of recombinant region is based on the core genome.
