# Supplementary material for: Neutral Genomic Microevolution of a Recently Emerged Pathogen, Salmonella enterica Serovar Agona
Source: PLoS Genet. 2013 Apr 18;9(4):e1003471. doi: 10.1371/journal.pgen.1003471 (PMC3630104; doi:10.1371/journal.pgen.1003471)
Supplement: Table S7 — Invariable IS elements in the core genome. (DOCX) [file pgen.1003471.s026.docx]

**Table S7** Invariable IS elements in the core genome

| **IS_ID** | **Best Hit** | **Family** | **Copy number** | **Position in SL483** |
| --- | --- | --- | --- | --- |
| ISN1 | ISSen1 | IS3 | 5 | 714171..715426; 1086220..1087475; 3279412..3280667; 3544776..3546031; 4678342..4679597 |
| ISN2 | IS1230B | IS3 | 3 | 351909..352124; 1122294..1123456; 4483803..4484686 |
| ISN3 | ISEhe3 | IS3 | 2 | 596779..596993; 2586486..2586839 |
| ISN4 | ISKpn8 | IS3 | 1 | 3756954..3757088 |
| ISN5 | ISYps8 | IS3 | 1 | 4488634..4488978 |
| ISN6 | IS1351 | IS3 | 1 | 4689626..4690737 |
| ISN7 | ISSoEn1 | IS5 | 1 | 471422..471712 |
| ISN8 | ISEc49 | IS66 | 1 | 316978..317262 |
| ISN9 | ISKpn2 | IS110 | 1 | 1991543..1992900 |
| ISN10 | ISEc41 | IS200/IS605 | 1 | 116194..117399 |
| ISN11 | IS285 | IS256 | 2 | 940258..941050; 4486626..4487159 |
| ISN12 | ISSoEn2 | IS256 | 1 | 329577..329708 |
| ISN13 | ISEc40 | IS630 | 1 | 2029918..2030073 |
| ISN14 | IS630 | IS630 | 1 | 3876778..3877026 |
| ISN15 | IS630 | IS630 | 1 | 4676478..4676978 |

**Note**: ISN1 and ISN3 are also present in the accessory genome (Table S8, S9).
