# Supplementary material for: Neutral Genomic Microevolution of a Recently Emerged Pathogen, Salmonella enterica Serovar Agona
Source: PLoS Genet. 2013 Apr 18;9(4):e1003471. doi: 10.1371/journal.pgen.1003471 (PMC3630104; doi:10.1371/journal.pgen.1003471)
Supplement: Table S8 — Variable IS insertions in the core genome. (DOCX) [file pgen.1003471.s027.docx]

**Table S8**. Variable IS insertions in the core genome

| **IS_ID** | **Best Hit** | **Family** | **Accquired Lineage** | **Insert Position in SL483** |
| --- | --- | --- | --- | --- |
| ISN1 | ISSen1 | IS3 | N26-N27 | 1989475..1990730 |
| ISN16 | ISVsa5 | IS4 | N25-N26 | 521414..522622 |
| ISN16 | ISVsa5 | IS4 | N25-N26 | 1556667..1557875 |
| ISN16 | ISVsa5 | IS4 | N25-N26 | 3105001..3106209 |
| ISN16 | ISVsa5 | IS4 | N26-N27 | 331418..332626 |
| ISN16 | ISVsa5 | IS4 | N27-25.H.03 | 2658206..2659414 |
| ISN16 | ISVsa5 | IS4 | N27-25.H.03 | 2975984..2977192 |
| ISN16 | ISVsa5 | IS4 | N27-25.H.03 | 4458764..4459972 |
| ISN17 | IS903B | IS5 | N05-64.H.00 | 4601344..4601354 |

**Note**: ISN17 is also present in plasmid P_2 (Table S9).
