# Supplementary material for: Neutral Genomic Microevolution of a Recently Emerged Pathogen, Salmonella enterica Serovar Agona
Source: PLoS Genet. 2013 Apr 18;9(4):e1003471. doi: 10.1371/journal.pgen.1003471 (PMC3630104; doi:10.1371/journal.pgen.1003471)
Supplement: Table S9 — IS elements in genomic islands or plasmids. (DOCX) [file pgen.1003471.s028.docx]

**Table S9.** IS elements in genomic islands or plasmids

| **IS_ID** | **Best Hit** | **Family** | **Genomic island/Plasmid: Lineage (deletion)** |
| --- | --- | --- | --- |
| ISN18 | IS1N | IS1 | P_2: N16-20.H.06 |
| ISN19 | IS1X3 | IS1 | P_2: N16-20.H.06; GI32: N16-21.H.10; GI24B: N05-62.H.72 |
| ISN3 | ISEhe3 | IS3 | GI26: N05-63.H.87 |
| ISN20 | IS1133 | IS3 | P_3A: N19-08.A.05 && N19-N20 && N20-01.O.05 (d) && N20-N21 (d) |
| ISN21 | ISEc15 | IS3 | P_2: N16-20.H.06 |
| ISN17 | IS903B | IS5 | P_2: N16-20.H.06 |
| ISN22 | ISEcl3 | IS5 | GI22: N32-39.O.03 (d) |
| ISN23 | IS5 | IS5 | P_2: N16-20.H.06; GI32: N16-21.H.10 |
| ISN24 | IS15 | IS6 | P_3A: N19-08.A.05 && N19-N20 && N20-01.O.05 (d) && N20-N21 (d); P_3C: N29-30.H.04; P_3D: N05-63.H.87 |
| ISN25 | IS1326 | IS21 | GI30C: N36-67.H.09 |
| ISN26 | IS100kyp | IS21 | P3_D: N05-63.H.87 |
| ISN27 | ISCro1 | IS66 | GI1A: N18-N23; P_3C: N29-30.H.04 |
| ISN28 | ISEc47 | IS66 | P_3B: N34-32.A.00 |
| ISN29 | ISCro1 | IS66 | GI24B: N05-62.H.72 |
| ISN30 | ISVsa3 | IS91 | GI30B: N25-24.H.04 |
| ISN31 | ISEc21 | IS110 | P_2: N16-20.H.06 |
| ISN32 | ISSen5 | IS630 | GI30A: N17-18.H.07 |
| ISN33 | ISCps6 | IS1182 | GI30C: N36-67.H.09 |
| ISN34 | ISPa38 | Tn3 | GI17: N33-34.H.09 |
| ISN35 | ISSba14 | Tn3 | P_4: N33-34.H.09 |
| ISN36 | ISYps3 | Tn3 | P_4: N33-34.H.09 |
| ISN37 | ISPa38 | Tn3 | GI21A: N05-N06 && N17-N18; GI5: N03-70.E.05; GI11A: N31-36.H.00; GI11B: N25-23.F.01 |
| ISN38 | ISSwi1 | Tn3 | P_3A: N19-08.A.05 && N19-N20 && N20-01.O.05 (d) && N20-N21 (d) |
| ISN39 | ISSba14 | Tn3 | P_2: N16-20.H.06 |
| ISN40 | ISPa38 | Tn3 | GI21B: N17-17.H.06 && N17-18.H.07 && N17-19.F.03; GI21C: N24-16.H.08; GI21F: N31-N32 && N32-39.O.03 (d); GI21G: N31-36.H.00 |
| ISN41 | ISShes11 | Tn3 | P_3B: N34-32.A.00; P_3C: N29-30.H.04 |
